# Interferon-Induced Transmembrane Protein 3 Is a Virus-Associated Protein Which Suppresses Porcine Reproductive and Respiratory Syndrome Virus Replication by Blocking Viral Membrane Fusion

**DOI:** 10.1128/JVI.01350-20

**Published:** 2020-11-23

**Authors:** Angke Zhang, Hong Duan, Huijun Zhao, Huancheng Liao, Yongkun Du, Liangliang Li, Dawei Jiang, Bo Wan, Yanan Wu, Pengchao Ji, En-Min Zhou, Gaiping Zhang

**Affiliations:** a College of Animal Science and Veterinary Medicine, Henan Agricultural University, Zhengzhou, Henan, China; b College of Veterinary Medicine, Northwest A&F University, Yangling, China; c Key Laboratory of Animal Immunology of the Ministry of Agriculture, Henan Academy of Agricultural Sciences, Zhengzhou, Henan, China; d College of Agronomy, Liaocheng University, Liaocheng, China; Loyola University Chicago

**Keywords:** IFITM3, PRRSV, cholesterol, cellular vesicles, membrane fusion

## Abstract

Porcine reproductive and respiratory syndrome virus (PRRSV) infection eliminates production of type I interferons (IFNs) in host cells, which triggers an antiviral immune response through the induction of downstream IFN-stimulated genes (ISGs), thus escaping the fate of host-mediated clearance. The IFN-induced transmembrane 3 (IFITM3) has recently been identified as an ISG and plays a pivotal role against enveloped RNA viruses by restricting cell entry. However, the role of IFITM3 in PRRSV replication is unknown. The present study demonstrated that overexpression of IFITM3 suppresses PRRSV replication, while silencing of endogenous IFITM3 prominently promoted PRRSV replication. Additionally, it was shown that IFITM3 undergoes S-palmitoylation and ubiquitination modification, and both posttranslational modifications contribute to the anti-PRRSV activity of IFITM3. Further study showed that PRRSV particles are transported into endosomes and then into lysosomes during the early stages of infection, and confocal microscopy results revealed that PRRSV particles are transported to IFITM3-positive cellular vesicles. By using a single virus particle fluorescent labeling technique, we confirmed that IFITM3 can restrict PRRSV membrane fusion by inducing accumulation of cholesterol in cellular vesicles. Additionally, we found that both endogenous and exogenous IFITM3 are incorporated into newly producing PRRS virions and diminish viral intrinsic infectivity. By using cell coculture systems, we found that IFITM3 effectively restricted PRRSV intercellular transmission, which may have been caused by disrupted membrane fusion and reduced viral infectivity. In conclusion, our results demonstrate, for the first time, that swine IFITM3 interferes with the life cycle of PRRSV, and possibly other enveloped arteritis viruses, at multiple steps.

**IMPORTANCE** Porcine reproductive and respiratory syndrome (PRRS), which is caused by PRRS virus (PRRSV), is of great economic significance to the swine industry. Due to the complicated immune escape mechanisms of PRRSV, there are no effective vaccines or therapeutic drugs currently available against PRRS. Identification of cellular factors and underlying mechanisms that establish an effective antiviral state against PRRSV can provide unique strategies for developing antiviral vaccines or drugs. As an interferon (IFN)-stimulated gene, the role of IFN-induced transmembrane 3 (IFITM3) in PRRSV infection has not been reported as of yet. In the present study, it was shown that IFITM3 can exert a potent anti-PRRSV effect, and PRRS virions are trafficked to IFITM3-containing cell vesicles, where viral membrane fusion is impaired by cholesterol accumulation that is induced by IFITM3. Additionally, both endogenous and exogenous IFITM3 are incorporated into newly assembled progeny virions, and this decreased their intrinsic infectivity.

## Abstract (precis)

Porcine reproductive and respiratory syndrome (PRRS), which is caused by PRRS virus (PRRSV), is of great economic significance to the swine industry. Due to the complicated immune escape mechanisms of PRRSV, there are no effective vaccines or therapeutic drugs currently available against PRRS. Identification of cellular factors and underlying mechanisms that establish an effective antiviral state against PRRSV can provide unique strategies for developing antiviral vaccines or drugs. As an interferon (IFN)-stimulated gene, the role of IFN-induced transmembrane 3 (IFITM3) in PRRSV infection has not been reported as of yet. In the present study, it was shown that IFITM3 can exert a potent anti-PRRSV effect, and PRRS virions are trafficked to IFITM3-containing cell vesicles, where viral membrane fusion is impaired by cholesterol accumulation that is induced by IFITM3. Additionally, both endogenous and exogenous IFITM3 are incorporated into newly assembled progeny virions, and this decreased their intrinsic infectivity.

## INTRODUCTION

Innate antiviral immunity is orchestrated by the interferon (IFN) system, which plays a pivotal role in early perception and confrontation of invading viruses by the mammalian host, and the interaction between the virus and the host IFN system largely determines the outcome of most viral diseases. IFN, whether endogenous or exogenous, induces the expression of a large number of IFN-stimulated genes (ISGs), the primary host effectors mediating the establishment of an antiviral status against viral infection. Hundreds of ISGs have been identified thus far, only a few of which have had their function characterized (1, 2). Understanding how individual ISGs restrict viral infection is of broad interest for designing antiviral therapeutics that can be targeted to specific or more general classes of pathogens.

The IFN-induced transmembrane proteins (IFITMs) are a family of ubiquitously expressed transmembrane proteins that respond differentially to IFN induction and viral infections. Humans have five IFITM proteins, IFITM1, 2, 3, 5, and 10 (3). IFITM1, 2, and 3 are expressed in a wide range of tissues, whereas IFITM5 expression appears to be limited to skeletal tissue, and the function of IFITM10 remains ambiguous (4). Thus, most studies have focused on IFITM1, 2, and 3. IFITM1, 2, and 3 have emerged as broad-acting restriction factors capable of disturbing the replication of multiple RNA viruses that enter the host cell through endocytosis, including influenza A virus (IAV), Dengue virus (DENV), West Nile virus, SARS coronavirus (SARS-CoV), and hepatitis C virus (5, 6). Although all three IFITM proteins are able to restrict viral replication to various degrees, IFITM3 alone mediates a large extent of the antiviral effects of IFN *in vitro* (7). IFITM3 is the most potent IFITM family member in restricting IAV replication *in vitro* (8, 9). Notably, IFITM3^−/−^ mice are more susceptible to IAV infection (10, 11). IFITM1, 2, and 3 have been shown to restrict Mycobacterium tuberculosis replication in human monocytes, while IFITM3 seems to play a central role in this process (12). For certain livestock viruses, IFITMs display antiviral effects as well. For example, IFITMs are reported to suppress replication of African swine fever virus, classical swine fever virus, and avian tembusu virus *in vitro* (13–15). However, some viruses are resistant to IFITM-mediated restriction. For example, IFITMs do not restrict infection of mouse leukemia virus, Machupo virus, Lassa virus, or lymphocytic choriomeningitis virus (16), highlighting the potential dual roles of IFITM3 in inhibiting viral replication.

The underlying mechanism by which IFITMs inhibit infection of a range of viruses, however, is still largely unknown. Accumulating evidence suggests that IFITMs may interfere with virus-endosome fusion to block enveloped virus entry (17–20). In mammalian cells, IFITM1 is primarily located in the plasma membrane, while IFITM2 and IFITM3 are predominantly localized to early and late endosomes and lysosomes, as shown by immunofluorescence and live-cell imaging studies (21). The cellular localization of the IFITMs may be a crucial determinant of their specificities toward viruses, as IFITM1 is more potent against viruses that enter through the plasma membrane or early endosomes, whereas IFITM2 and IFITM3 are more potent against viruses that enter cells through late endosomal compartments. Effective restriction of viruses that enter from the late endosome, such as IAV, Ebola virus, and SARS-CoV seems consistent with the cellular localization of IFITM2 and IFITM3 proteins (16). Indeed, mutation of IFITM3 that redistributes the late endosome/lysosome-resident protein to the cytoplasmic membrane abolishes its antiviral activity against IAV (22). However, IFITMs also restrict vesicular stomatitis virus, which appear to fuse with early endosomes (9). As for the mechanism associated with the inhibition of membrane fusion, IFITMs have been reported to curtail viral infection, in part by resulting in the accumulation of cholesterol in late endosomes as a result of IFITM-mediated disruption of the interaction between the vesicle membrane-protein-associated protein A (VAPA) and oxysterol binding protein (OSBP) (23). A recent study provided evidence of the antiviral effect of cholesterol accumulation in late endosomes/lysosomes and confirmed accumulation of cholesterol in these membrane-associated compartments upon IFITM3 expression (13, 23). Due to the important role of lipids in membrane fusion, these findings offer an attractive paradigm for a broad antiviral defense mechanism that involves altering the lipid composition of cellular membranes. Yount et al. recently showed that human IFITM3 undergoes both ubiquitination and S-palmitoylation modification in cells and that these posttranslational modifications strikingly regulate IFITM3 cellular localization and its anti-influenza activities (24). In addition to the aforementioned antiviral mechanisms, certain studies have shown that IFITMs abolished viral replication through merging with the viral envelope protein during virion assembly and are eventually incorporated into newly produced particles that display decreased infectivity compared with their wild-type (WT) counterparts (25, 26).

Porcine reproductive and respiratory syndrome (PRRS), caused by PRRS virus (PRRSV), is characterized by reproductive failure in sows and respiratory diseases in pigs of all ages and results in huge economic losses to the swine industry worldwide (27). PRRSV is an enveloped, single-stranded RNA virus which belongs to the genus *Porartevirus*, family *Arteriviridae*, and order *Nidovirales* (28, 29). PRRSV has highly restricted cell tropism whereby it infects the monocyte-macrophage lineage cells, including porcine alveolar macrophages (PAMs), the primary target of PRRSV infection *in vivo* (30, 31). The African green monkey kidney cell line MA-104 and its subclone Marc-145 are also susceptible to PRRSV infection and have been frequently used in PRRSV studies *in vitro* (32, 33). Although the mechanism of PRRSV particle entry into host cells remains to be fully understood, it has been shown that the envelope glycoproteins (GP3, GP4, GP5, and M) on the surface of the viral envelop regulate cell entry via binding to cell surface receptors, such as scavenger receptor (CD163), sialoadhesin (Sn), and heparan sulfate (HS) among others (34–36). After receptor clathrin-dependent endocytosis, the endocytosed virions further undergo low-pH-dependent viral membrane fusion, which leads to the nucleocapsid disintegration and the release of viral genomes into the cytoplasm, initiating its replication process. Previous studies concluded that after entering the host cells, PRRSV particles are transported to early endosomes without being further transported to acidified late endosomes and lysosomes (37), exhibiting an intracellular transport pathway that appears to be less consistent with other enveloped RNA viruses. Thus far, the effect of IFITM3 on PRRSV replication and the relevant mechanism is unknown, to the best of our knowledge. Since the antiviral activity of IFITM3 is likely mediated by preventing endosome fusion and subsequent viral entry into the cytosol (19, 38), or by leading to the production of virions with reduced infectivity, we hypothesize that swine IFITM3 is able to abrogate PRRSV replication by preventing virions escaping from the endocytic pathway during an early stage of the life cycle and by incorporation into the envelope of newly produced PRRS virions that lead to decreased infectivity.

The results of the present study show that both endogenous and exogenous IFITM3 serves an important role in prohibiting PRRSV replication, and S-palmitoylation and ubiquitination modifications benefit both anti-PRRSV activities of IFITM3. Furthermore, IFITM3 is essential in mediating antiviral activity of IFN-α. Further study suggests that PRRSV particles are first transported into early endosomes and then into late endosomes and lysosomes, which is inconsistent with previous reports that the virus entered only the early endosomes but not late endosomes or lysosomes in the early stage of infection. By using single virus particle fluorescent labeling, we demonstrate that IFITM3 restricts PRRSV membrane fusion by disturbing intracellular cholesterol homeostasis. Both exogenous and endogenous IFITM3 are incorporated into virions and reduce viral infectivity, and intercellular transmission of PRRSV is abolished by IFITM3 as well. In conclusion, the results of the present study are the first to demonstrate that IFITM3 restricts replication of one member of the arteritis virus family PRRSV that utilizes endosome-dependent cell entry mechanisms. Additionally, IFITM3 interferes with PRRSV replication by resulting in the production of virions with reduced infectivity. The dual inhibitory effect also emphasizes the importance of IFITM3 in the host anti-PRRSV response.

## RESULTS

### IFITM3 inhibits PRRSV replication *in vitro*.

**FIG 1:**
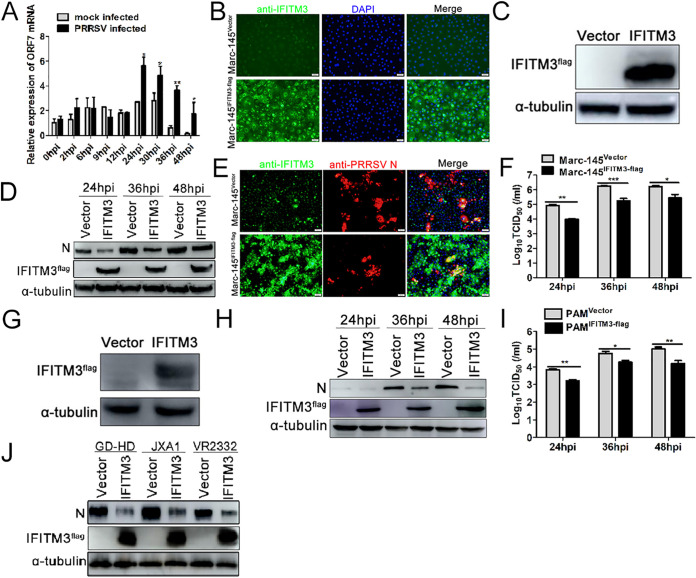
Overexpression of IFITM3 suppresses PRRSV replication *in vitro*. (A) Expression of IFITM3 mRNA in PRRSV-infected PAMs. (B) IFA or (C) Western blotting verification of stable expression of IFITM3 in Marc-145 cells. Marc-145-Vector or Marc-145-IFITM3-flag cells were infected with PRRSV (0.1 MOI) at 37°C for 1 h, and cells were subsequently maintained in 3% FBS+DMEM. (D and F) Cells were collected after 24, 36, and 48 hpi for detection of IFITM3 or N protein expression using (D) Western blotting, and supernatant virus titers using (F) TCID_50_. (E) A portion of cells were fixed using 70% 20°C prechilled alcohol 24 hpi for IFA. (G) PAMs were transduced with recombinant lentivirus expressing IFITM3-flag or control lentivirus for 36 h, and cells were harvested for IFITM3 analysis. (H and I) PAMs transduced with recombinant lentivirus expressing IFITM3-flag or control lentivirus were infected with 0.1 MOI of PRRSV, and cells and supernatants were collected at 24, 36, and 48 hpi to determine (H) IFITM3 and N protein expression and (I) supernatant virus titers. (J) Marc-145 cells were infected with GD-HD-, JXA1-, and VR2332-PRRSV (0.1 MOI). At 48 hpi, cells were harvested and analyzed by Western blotting. IFA, indirect immunofluorescence assay; MOI, multiplicity of infection; hpi, hours postinfection; PAM, porcine alveolar macrophage; IFITM, interferon-induced transmembrane; PRRSV, porcine reproductive and respiratory syndrome virus.

To examine the expression of IFITM3 following PRRSV infection, PAMs were infected or mock infected with 0.1 multiplicity of infection (MOI) PRRSV, and IFITM3 was detected using reverse transcriptase quantitative PCR (RT-qPCR). The results showed that PRRSV infection induced IFITM3 expression in PAMs (Fig. 1A). To understand the role of IFITM3 on PRRSV replication, recombinant cell lines stably expressing swine IFITM3 were first established. As shown in Fig. 1B, expression of IFITM3 in Marc-145-IFITM3-flag cells (lower panel) was notably overexpressed compared with the Marc-145-Vector cell line (upper panel). Western blotting using an anti-flag antibody further confirmed the overexpression of IFITM3 protein (Fig. 1C), suggesting that the recombinant cell line was successfully established.

Subsequently, the effect of IFITM3 on PRRSV infection was assessed. According to the Western blotting and immunofluorescence assay (IFA) results, expression of PRRSV N protein was notably decreased compared with the control group after 24, 36, and 48 hours postinfection (hpi) (Fig. 1D and E). Overexpression of IFITM3 markedly decreased supernatant virus titers as well (Fig. 1F). As the natural host cell of PRRSV, the effect of IFITM3 on PRRSV replication in PAMs was also determined. Consistently, overexpression of IFITM3 reduced N protein expression in PAMs compared with the control group (Fig. 1G and H) and simultaneously decreased supernatant virus titers (Fig. 1I). In addition, Western blotting results suggested that IFITM3 suppressed GD-HD-, JXA1-, and VR2332-PRRSV N protein expression, suggesting that IFITM3 suppression of PRRSV infection was not strain dependent (Fig. 1J). Collectively, these results suggest that overexpression of IFITM3 can inhibit PRRSV replication *in vitro*.

### PRRSV particles traffic along the endosome-lysosome pathway.

**FIG 2:**
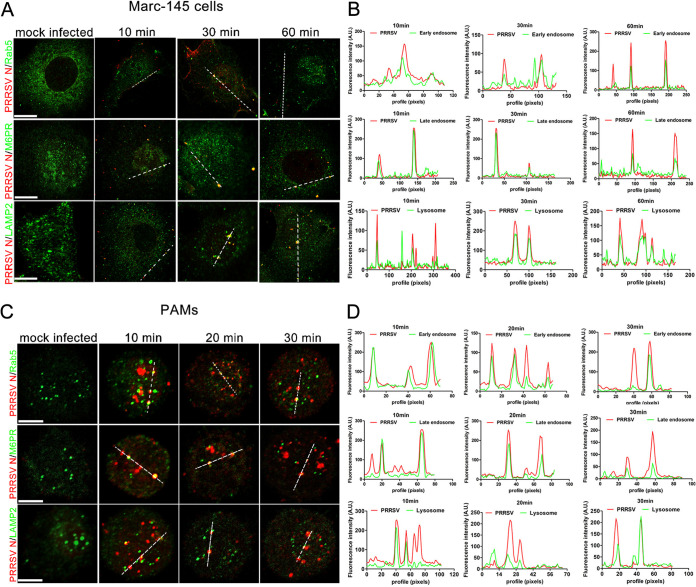
PRRSV particles are transported along the endosome-lysosome pathway during an early stage of infection. Marc-145 cells or PAMs were prechilled on ice for 30 min before incubation with 1.0 MOI of PRRSV. Cells were further incubated on ice for 1 h to ensure abundant adsorption of virus. Subsequently, cells were washed extensively with ice-cold PBS to remove unabsorbed virus and incubated at 37°C for the indicated time points. Cells were fixed with 70% −20°C prechilled alcohol, and cellular compartments and viral proteins were stained with specific antibodies against early endosomes (Rab5), late endosomes (M6PR), lysosomes (LAMP2), and capsid protein (N protein), followed by staining with Alexa Fluor 488-conjugated goat anti-rabbit IgG (H&L) antibody to visualize cellular compartments (green) and Alexa Fluor-594 conjugated goat anti-mouse IgG (H&L) antibody to visualize virions (red). Fluorescent images were acquired with a confocal laser scanning microscope. Scale bar, 10 μm. (A and C) Confocal immunofluorescence images showing PRRSV particles colocalized with endosomes and lysosomes at different time points. (B and D) Colocalization analysis corresponding to the PRRSV particles and cellular compartments. PAM, porcine alveolar macrophage; PRRSV, porcine reproductive and respiratory syndrome virus; MOI, multiplicity of infection.

The intracellular transportation of PRRSV particles was first investigated to determine on which stage of the PRRSV replication cycle IFITM3 exerted its antiviral effects. Colocalization was defined as any observed merge of the red fluorescence of viral protein with the green fluorescence of cell organelles. As shown in Fig. 2A, colocalization was observed between PRRSV particles and early/late endosomes or lysosomes in Marc-145 cells. To further confirm that PRRSV required early/late endosomes and lysosomes for transportation, subcellular localization of PRRS virions was analyzed in PAMs as well as the virus’ *in vivo* target cells. Confocal results showed that PRRS virions occurred in early/late endosomes and lysosomes similar to the Marc-145 cells (Fig. 2C), and colocalization analysis results further confirmed the above results (Fig. 2B and D). The red spots at the periphery of the cells represent viruses that were sticking to the cell but were not yet internalized (Fig. 2A and C). Previous reports showed that PRRSV particles were trafficked only to early endosomes but not to late endosomes and lysosomes for productive infection (37). The above results suggest that PRRS virions are trafficked along the endosome-lysosome pathway, similar to other enveloped viruses. As PRRSV particles undergo a similar transport process within both Marc-145 cells and PAMs, Marc-145 cells were selected as the research model for the subsequent experiments to examine the underlying molecular mechanism of anti-PRRSV activity exerted by IFITM3, unless otherwise indicated.

### IFITM3 does not block PRRSV attachment, entry, or access to cell vesicles.

**FIG 3:**
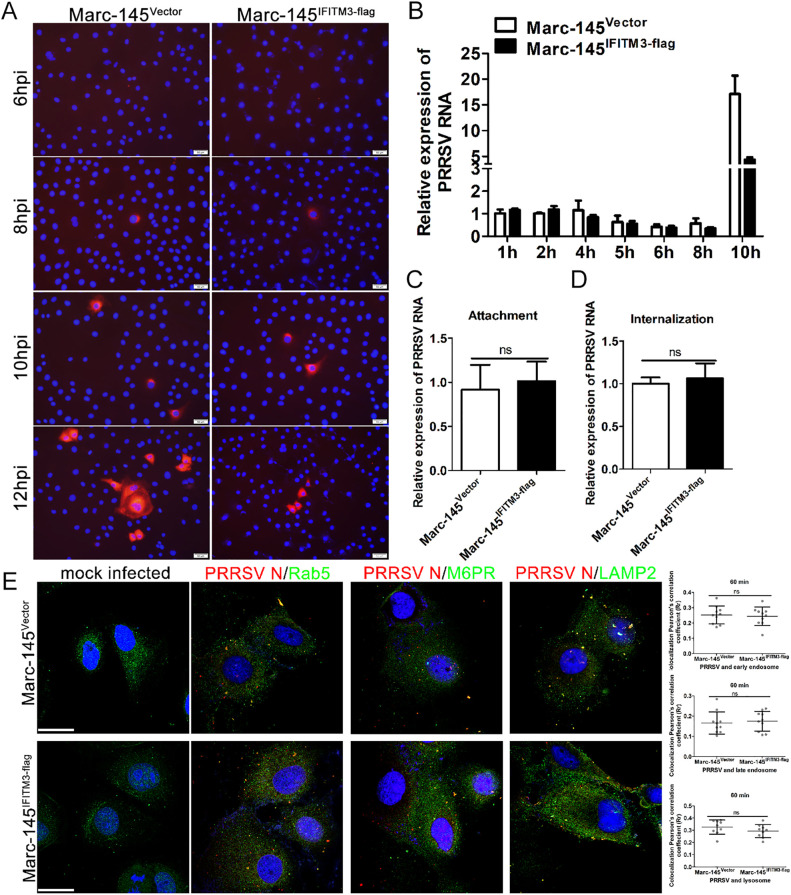
IFITM3 does not affect PRRSV particle attachment, entry, or access to endosomes or lysosomes. (A) Marc-145-Vector or Marc-145-IFITM3-flag cells were incubated with PRRSV at an MOI of 1.0 for 6, 8, 10, and 12 h. The infected cells were fixed and stained with anti-PRRSV N antibody and counterstained with DAPI to visualize the nuclei. (B) Marc-145-Vector or Marc-145-IFITM3-flag cells were infected with 1.0 MOI of PRRSV for 1 h at 37°C, and then cells were washed with PBS and cultured with 3% FBS+DMEM. Cells were harvested 1, 2, 4, 5, 6, 8, or 10 hpi, and PRRSV genome levels were determined. Prechilled Marc-145-Vector or Marc-145-IFITM3-flag cells were infected with PRRSV (1.0 MOI) and further chilled on ice for 1 h. (C) For the attachment assay, after washing 3 times using ice-cold PBS, cells were harvested for PRRSV genome abundance analysis. (D) For the entry assay, cells were washed with PBS three times and treated with 37°C prewarmed DMEM and incubated at 37°C for 1 h, followed by trypsin treatment for 30 sec and 3 washes with PBS. Cells were collected for PRRSV genome content analysis. (E) Marc-145-Vector or Marc-145-IFITM3-flag cells were inoculated with 1.0 MOI PRRSV for 1 h at 37°C, and then cells were fixed and stained with anti-PRRSV N, -Rab5, -M6PR, and -LAMP2 antibodies, followed by an Alexa Fluor 488-conjugated goat anti-rabbit IgG (H&L) antibody and an Alexa Fluor 594-conjugated goat anti-mouse IgG (H&L) antibody. Colocalization of PRRSV particles with cellular vesicles was quantified, and representative images are presented. Scale bar, 10 μm. Horizontal bars represent the mean of the Pearson’s correlation coefficient (Rr) calculated based on 10 fields of view, with error bars marking the 95% confidence intervals. PRRSV, porcine reproductive and respiratory syndrome virus; MOI, multiplicity of infection; hpi, hours postinfection.

To determine the stage of the PRRSV life cycle that was restricted by IFITM3, we first characterized the kinetics of the viral N protein and NSP2 gene expression during the course of PRRSV infection. As shown in Fig. 3A, IFA results indicated that viral N protein was initially detected at ∼8 hpi. RT-qPCR results showed that NSP2 gene levels began to increase from ∼8 hpi (Fig. 3B), consistent with the IFA results, suggesting that at this time point, viral transcription and replication were already under way, and the replication cycle of PRRSV is ∼8 h. Compared with the viral replication kinetics in Marc-145-Vector cells, both N protein and NSP2 gene levels began to lower in Marc-145-IFITM3-flag cells compared with control cells from 8 h onward, and the gap gradually expanded, as illustrated by the IFA and RT-qPCR results (Fig. 3A and B). Taken together, these results suggest that IFITM3 exerted its antiviral effects during a single viral replication cycle.

To further investigate at which stage of the single cycle of PRRSV replication IFITM3 exerted its antiviral effects, the effect of IFITM3 on PRRSV attachment and internalization was assessed. RT-qPCR results of attachment and entry assay both showed that there was no significant difference in the amount of viral RNA associated with control or IFITM3-overexpressing cells (Fig. 3C and D), indicating that IFITM3 does not modulate viral attachment and internalization into cells. Subsequently, whether IFITM3 prevented PRRSV particles from translocating to endosomes and lysosomes was investigated using confocal analysis. A total of 10 different fields of view were selected for each sample, and colocalization analysis was performed using ImageJ; representative images are presented in Fig. 3E. Confocal analysis results indicated that overexpression of IFITM3 did not block PRRS virion access to acidic endosomes or lysosomes (Fig. 3E). Taken together, these results indicate that IFITM3 does not block PRRSV attachment, entry, or access to endosomes or lysosomes during the early stages of replication.

### Both PRRS virions and IFITM3 colocalize with endosomes and lysosomes.

**FIG 4:**
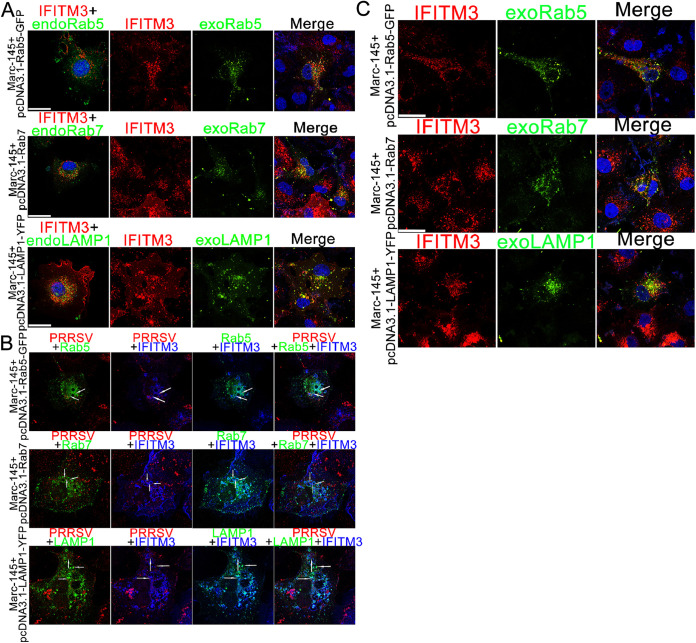
PRRSV particles are transported to IFITM3-containing endosomes or lysosomes. (A) Marc-145-IFITM3-flag cells transfected with pcDNA3.1-Rab5-GFP, -Rab7, and -LAMP1-YFP plasmids for 48 h were fixed with 4% paraformaldehyde and permeabilized with 0.3% Triton X-100. Cells were labeled with anti-IFITM3 and -Rab7 antibodies followed by Alexa Fluor 594-conjugated goat anti-mouse IgG (H&L) antibody for IFITM3 (red) and Alexa Fluor 488-conjugated goat anti-rabbit IgG (H&L) antibody for Rab7 (green), and cell nuclei were counterstained using DAPI. Rab5 and LAMP1 (green) were observed directly using a confocal microscope. (B) PRRSV were allowed to bind to prechilled Marc-145-IFITM3-flag cells for 1 h on ice, unbound virions were washed, and cells were warmed to 37°C for 45 min. IFITM3 was stained using an anti-IFITM3 antibody and the corresponding fluorescent second antibody (blue), and PRRSV was stained using an anti-N antibody and a fluorescent second antibody (red). (C) Marc-145-IFITM3-flag cells were infected with 0.1 MOI of PRRSV for 12 h, and then cells were transfected with pcDNA3.1-Rab5-GFP, -Rab7, and -LAMP1-YFP plasmids for 36 h and fixed with 4% paraformaldehyde and permeabilized with 0.3% Triton X-100. Cells were labeled as described in Fig. 4A. Scale bar, 10 μM. PRRSV, porcine reproductive and respiratory syndrome virus; IFITM, interferon-induced transmembrane.

Since our results showed that PRRSV was transported to endosomes and lysosomes at the beginning of infection (Fig. 2), the subcellular localization of IFITM3 was further determined. Marc-145-IFITM3-flag cells were transiently transfected with pcDNA3.1-Rab5-GFP, pcDNA3.1-Rab7, and pcDNA3.1-LAMP1-YFP plasmids, Rab7 was labeled with an anti-Rab7 antibody, and IFITM3 was labeled with an anti-IFITM3 antibody. Colocalization analysis results showed that the vast majority of IFITM3 colocalized with early/late endosomes and lysosomes (Fig. 4A), and PRRSV infection did not change the subcellular distribution of IFITM3 (Fig. 4C). We next investigated whether internalized PRRSV particles were transported to IFITM3-positive endosomes or lysosomes. PRRSV was first bound and allowed to enter the Marc-145-IFITM3-flag cells by incubating at 37°C for 45 min. Cells were fixed and labeled for N and IFITM3-flag proteins and visualized by confocal microscopy. Representative images of PRRSV particle colocalization with IFITM3-containing vesicles are presented in Fig. 4B. These data suggest that endocytosed PRRSV particles are delivered to IFITM3-positive endosomes and lysosomes in the initial stages of infection.

### IFITM3 restricts membrane fusion between PRRSV and cell vesicles.

**FIG 5:**
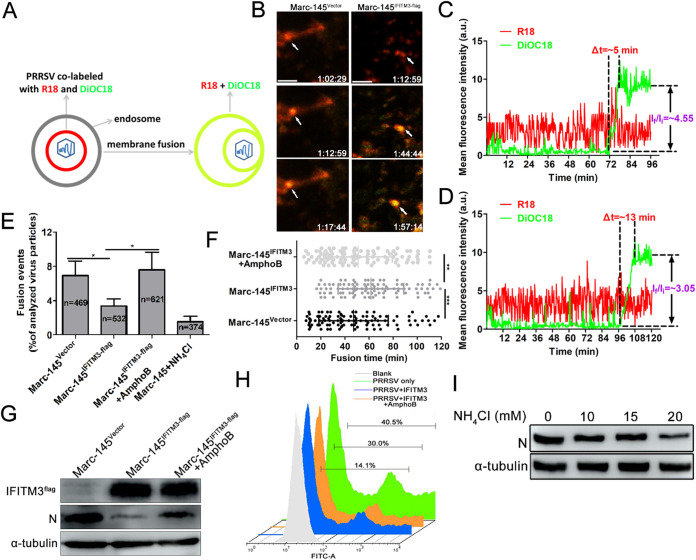
IFITM3 restricts PRRSV membrane fusion. (A) Schematic illustration of the single-virus fusion assay. PRRS virions were colabeled with DiOC18 (green) and R18 (red). In this model, viral membrane fusion led to a decrease of DiOC18 concentration, which appeared as a sudden increase of the green signal (dequenching), while the red fluorescence intensity remained almost unchanged. PRRSV particles colabeled with DiOC18 and R18 were prebound to Marc-145-Vector or Marc-145-IFITM3-flag cells for 1 h on ice and then incubated at 37°C for 2 h. Particle fusion with endosomes or lysosomes (white arrows) exhibited a marked increase in green signal and thus appeared yellow. (B) Time-lapse images from the single PRRSV fusion event showed the increase of green signal at around 72 min in Marc-145-Vector cells and 104 min in Marc-145-IFITM3-flag cells, indicating the fusion events. Scale bar, 4 μm. (C and D) Particle fluorescence intensities obtained by tracking virions in Marc-145-Vector or Marc-145-IFITM3-flag cells. Δt represents the time for complete dequenching. (E) Single PRRSV fusion efficiency in Marc-145-Vector or Marc-145-IFITM3-flag cells. (F) PRRSV fusion events following synchronized infection in Marc-145-Vector or Marc-145-IFITM3-flag cells (100 dequenching particles tracked in 5 independent experiments). (G and H) Marc-145-IFITM3-flag cells were pretreated with AmphoB (1 μM) for 1 h and then infected with 0.1 MOI of GFP-PRRSV. N protein- and GFP-PRRSV-positive cells were analyzed using Western blotting and FACS, respectively. (I) Marc-145 cells were infected with 0.1 MOI of PRRSV for 1 h on ice, and then cells were treated with 10, 15, and 20 mM NH_4_Cl for 36 h. N protein was analyzed using Western blotting. PRRSV, porcine reproductive and respiratory syndrome virus; IFITM, interferon-induced transmembrane; MOI, multiplicity of infection; DiOC18, 3,3′-dioctadecyloxacarbocyanine; R18, octadecyl rhodamine B.

To further investigate the molecular mechanism of IFITM3 inhibition of PRRSV infection, a direct virus-cell fusion assay was employed to evaluate the extent of restriction. To visualize single PRRSV-endosome fusion under a fluorescence microscope in Marc-145 cells, the PRRSV lipid membrane was labeled with two lipophilic probes, DiOC18 and R18. In the labeled virus, DiOC18 probes are incorporated into viral membranes at high concentrations, and this leads to suppression of the green fluorescence to a level similar to that of the red fluorescence by both self-quenching of DiOC18 and fluorescent resonance energy transfer from DiOC18 to R18. Virus-endosome fusion decreases the density of the green probe and consequently increases the dequenching of the fluorescence of the probe (Fig. 5A). The green (488-nm) and red (594-nm) fluorescence images of cells were obtained simultaneously using two detectors on a confocal microscope. Time-lapse images showed the initial trafficking of a representative DiOC18/R18 labeled virus in Marc-145-Vector cells, until fusion occurred, as evidenced by the enhancement of green fluorescence, while the red signal remained relatively constant (Fig. 5B and C). Importantly, PRRSV membrane fusion was also readily detected in Marc-145-IFITM3-flag cells (Fig. 5B and D). From these traces, the time required for complete dequenching (Δt) and the extent of dequenching (ratio of the initial and final mean intensities I_f_/I_i_) can be determined; Δt was 5 min in Marc-145-Vector cells, which was shorter than the Marc-145-IFITM3-flag cells (13 min; Fig. 5C and D). Considering that PRRS virions colocalized with IFITM3-positive endosomes and lysosomes (Fig. 4), we hypothesized that a sufficiently high local IFITM3 concentration was required for restriction of PRRSV fusion. To test this hypothesis, the number of virus particles that underwent membrane fusion events in both Marc-145-Vector and Marc-145-IFITM3-flag cells were counted for as long as the time-lapse imaging was performed. Analysis of the 532 particles revealed that only 18 single viral fusion events (3.38%) occurred after extensive PRRS virion colocalization with IFITM3-positive endosomes or lysosomes (Fig. 5E), while 32 single viral fusion events (6.82%) were observed in a total of 469 analyzed particles in Marc-145-Vector cells. Redistribution of DiOC18 was mediated by low pH-dependent virus-endosome fusion, as evidenced by potent inhibition of membrane fusion by NH_4_Cl treatment (1.53%; Fig. 5E), while AmphoB, which destabilizes lipid membranes, prominently reversed inhibition of membrane fusion elicited by IFITM3 (7.62%; Fig. 5E). The rate at which PRRSV particles underwent membrane fusion was longer in Marc-145-IFITM3-flag cells than in Marc-145-Vector cells (Fig. 5F), with a t_1/2_ of approximately 62 min for the former, and 47 min for the latter. In AmphoB-treated Marc-145-IFITM3-flag cells, the DiOC18-PRRSV dequenching rate was quicker than that of untreated cells (Fig. 5F), and the t_1/2_ for fusion occurred ∼10 min earlier, even though virus trafficking times may vary widely even in synchronized infections (Fig. 5F). Additionally, AmphoB partially reversed the inhibitory effect of IFITM3 on PRRSV replication, as shown by the Western blot and fluorescence-activated cell sorter (FACS) results (Fig. 5G and H). Inhibition of intracellular vesicle acidification can also inhibit PRRSV replication, since concentration-dependent NH_4_Cl suppressed PRRSV replication in Marc-145 cells (Fig. 5I). Together, these findings suggest that the mechanism of IFITM3-mediated restriction arises likely from the disruption of membrane fusion required for PRRSV vesicle transport.

### IFITM3-induced endosome/lysosome cholesterol accumulation impairs PRRSV membrane fusion.

**FIG 6:**
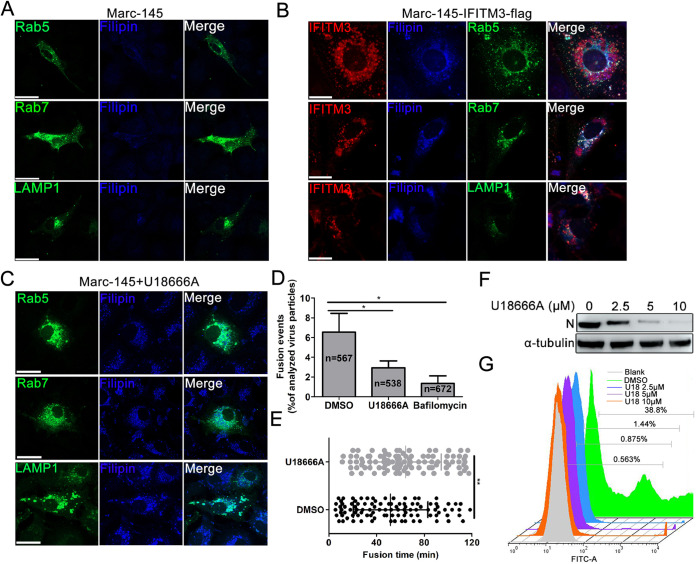
Accumulation of cholesterol in cell vesicles induced by IFITM3 effectively impairs viral membrane fusion. (A) Cholesterol distribution in Marc-145 cells transfected with pcDNA3.1-Rab5-GFP, -Rab7, and -LAMP1-YFP plasmids. (B) Marc-145-IFITM3-flag cells were transfected with pcDNA3.1-Rab5-GFP, -Rab7, and -LAMP1-YFP at 37°C for 48 h followed by fixing, permeabilizing, and labeling with anti-IFITM3 and -Rab7 antibodies and the corresponding fluorescent secondary antibodies. Subsequently, cells were stained using 50 μg/ml filipin for 2 h at room temperature in the dark for detection of cholesterol. Images showing colocalization between IFITM3, endosomes, lysosomes, and cholesterol were obtained using a confocal microscope. (C) Marc-145 cells transfected with pcDNA3.1-Rab5-GFP, -Rab7, and -LAMP1-YFP for 36 h were treated with 10 μM U18666A for 12 h, and then cells were fixed and endogenous cholesterol was stained using 50 μg/ml filipin in the dark for 2 h at room temperature. Scale bar, 10 μm. (D) Disturbance of intracellular cholesterol efflux reduced PRRSV membrane fusion. R18 and DiOC18 double labeled PRRS virions were prebound in the cold to DMSO- and U18666A-treated Marc-145 cells, and virus entry was initiated by transfer to 37°C for 2 h. Control experiments were performed by pretreating Marc-145 cells with 10 μM BafA1 for 1 h. Viral fusion events were normalized to the total number of cell-bound particles from 10 independent experiments. (E) PRRSV fusion events following synchronized infection of DMSO-treated or 10 μM U18666A-treated Marc-145 cells (100 dequenching particles tracked in 10 independent experiments). (F and G) Marc-145 cells were pretreated with 0, 2.5, 5, and 10 μM U18666A followed by inoculating with 0.1 MOI of GFP-PRRSV for 1 h at 37°C. Cells were collected 36 hpi for GFP-PRRSV-positive cell analysis using FACS, and for N protein expression detection using Western blotting. PRRSV, porcine reproductive and respiratory syndrome virus; IFITM, interferon-induced transmembrane; MOI, multiplicity of infection; hpi, hours postinfection; DiOC18, 3,3′-dioctadecyloxacarbocyanine; R18, octadecyl rhodamine B.

A recent study has shown that IFITM3 caused cholesterol accumulation in late endosomes through disrupting the interaction between VAPA and OSBP (23). Change in endosomal distribution is a characteristic phenotype of altered cholesterol endosomal efflux, which is required for correct endosomal function. Thus, we speculated that IFITM3 might cause accumulation of cholesterol in endosomes or lysosomes, which results in obstruction of PRRSV fusion. Marc-145-Vector/-IFITM3-flag cells transfected with Rab5, Rab7, or LAMP1 expression plasmids were incubated with filipin for detection of intracellular cholesterol levels. Confocal analysis results showed that cholesterol was diffusely distributed in the plasma membrane, cytoplasm, and endosomes in Marc-145-Vector cells (Fig. 6A), while intense intracellular cholesterol accumulation was observed at the perinuclear area, and cholesterol also colocalized with the IFITM3-positive endosomes and lysosome vesicles in Marc-145-IFITM3-flag cells (Fig. 6B). These findings indicate that IFITM3 overexpression results in cholesterol accumulation in endosomal and lysosomal compartments.

To further determine whether cholesterol accumulation in cell compartments was correlated with PRRSV fusion, U18666A, an amphipathic steroid which is widely used to block intracellular trafficking of cholesterol (39), was utilized to evaluate its effect on viral fusion. First, the subcellular distribution of cholesterol after U18666A treatment was detected in Marc-145 cells. Confocal results showed that in the presence of U18666A, cholesterol accumulated in the perinuclear swollen early/late endosomes and lysosomes (Fig. 6C). To evaluate the effect of cholesterol on PRRSV membrane fusion, viral envelope-endosome fusion assay utilizing DiOC18 and R18 labeled PRRSV was employed to identify potential fusion defects. U18666A treatment-induced cholesterol accumulation resulted in a pronounced decrease in DiOC18 dequenching compared with the DMSO treatment group (6.55% versus 2.93%; Fig. 6D), indicating that PRRSV membrane fusion within cholesterol-laden endosomes or lysosomes was impaired. BafA1, an inhibitor of the vacuolar ATPase required for endosomal acidification, which can block endosomal acidification, an essential prerequisite for successful PRRSV membrane fusion, prominently abolished DiOC18 dequenching (6.55% versus 1.35%; Fig. 6D). To further clarify the effect of vesicle cholesterol on viral fusion, dequenching rates of DiOC18 were analyzed. The DiOC18-PRRSV dequenching rate was delayed in U18666A-treated Marc-145 cells compared with untreated cells, with a t_1/2_ of ∼11 min longer than that of untreated cells (Fig. 6E). The effect of U18666A treatment on PRRSV infection in Marc-145 cells was subsequently determined. Western blotting results showed that U18666A suppressed PRRSV N protein expression in a concentration-dependent manner (Fig. 6F), and FACS analysis of GFP-PRRSV-positive cells also showed similar results (Fig. 6G), indicating that cholesterol accumulation-induced membrane fusion disorder effectively impeded PRRSV replication.

### Both S-palmitoylation and ubiquitination of the IFITM3 protein are critical for anti-PRRSV activity.

**FIG 7:**
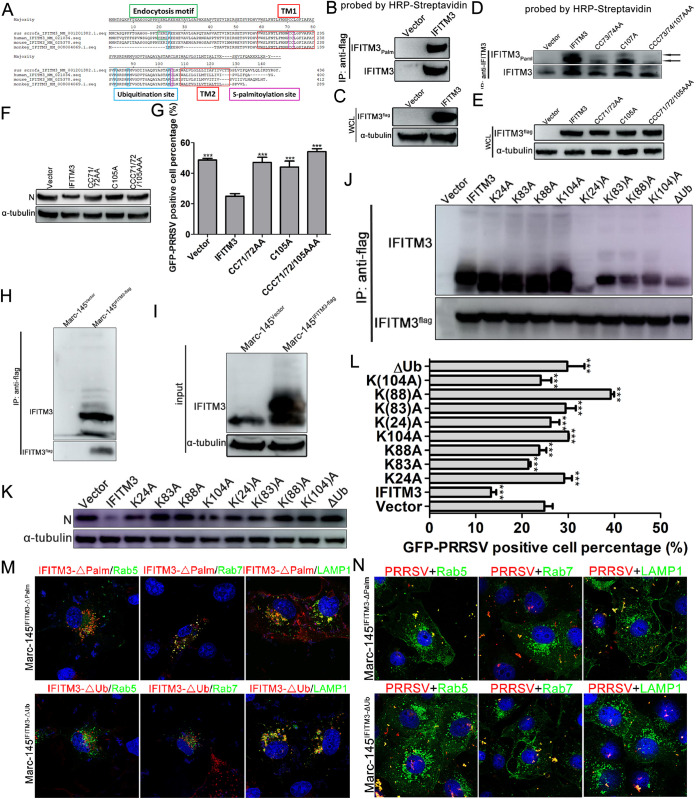
Both S-palmitoylation and ubiquitination of IFITM3 are critical for inhibition of PRRSV infection. (A) Alignment of human, mouse, porcine, and monkey IFITM3 using the ClustalV method. (B) IFITM3 palmitoylation was detected in Marc-145-Vector or Marc-145-IFITM3-flag cells using streptavidin-HRP following an ABE assay and immunoprecipitation with anti-flag MagBeads. A portion of the immunoprecipitation products was probed with an anti-IFITM3 antibody. (C) Another portion of cell lysates served as input. (D) Detection of palmitoylation of IFITM3 and its CC71/72AA, C105A, and CCC71/72/105AAA mutants in Marc-145-IFITM3-flag cells using streptavidin-HRP following an ABE assay and immunoprecipitation with anti-IFITM3 MagBeads. A portion of the immunoprecipitation products was probed with anti-IFITM3 antibody. (E) Another portion of cell lysates served as input. (F and G) Marc-145-Vector, Marc-145-IFITM3-flag cells, and their corresponding mutants were infected with 0.1 MOI GFP-PRRSV, and cells were harvested at 36 hpi for Western blotting and FACS analysis. (H) IFITM3 ubiquitination was determined using anti-flag MagBeads followed by Western blotting detection using anti-IFITM3 antibody. (I) A portion of cell lysates served as input. (J) Immunoprecipitation of IFITM3 and its K24A, K83A, K88A, K104A, K(24)A, K(83)A, K(88)A, K(104)A, and ΔUb mutants in recombinant cells was performed using anti-flag MagBeads, and then Western blotting was performed using anti-IFITM3 antibody. (K and L) Marc-145-Vector, Marc-145-IFITM3-flag, and their corresponding mutants infected with 0.1 MOI GFP-PRRSV were collected at 36 hpi and subjected to Western blotting or FACS detection. (M) Marc-145-IFITM3-ΔPal-flag or Marc-145-IFITM3-ΔUb-flag cells were transfected with pcDNA3.1-Rab5-GFP, -Rab7, or -LAMP1-YFP plasmids followed by staining with anti-Rab7 antibody 48 h posttransfection. Fluorescent images were acquired with a confocal laser scanning microscope. Scale bar, 10 μm. (N) Marc-145-IFITM3-ΔPal-flag or Marc-145-IFITM3-ΔUb-flag cells were transfected with pcDNA3.1-Rab5-GFP, -Rab7, or -LAMP1-YFP plasmids followed by inoculation with 1.0 MOI PRRSV for 1 h at 37°C, and then cells were fixed and stained with anti-PRRSV N antibody and Alexa Fluor 594-conjugated goat anti-mouse IgG (H&L) antibody. Scale bar, 10 μm. PRRSV, porcine reproductive and respiratory syndrome virus; IFITM, interferon-induced transmembrane; MOI, multiplicity of infection; HRP, horseradish peroxidase; ABE, acyl-biotin exchange; hpi, hours postinfection; for K(24)A, K(83)A, K(88)A, K(104)A, the numbers is parentheses indicate the one lysine of four that is not mutated to alanine.

To determine whether swine IFITM3 was S-palmitoylated and the role of S-palmitoylation in its anti-PRRSV activity, amino acid sequence analysis was performed. The amino acid alignment of the IFITM3 from human, pig, and monkey showed that IFITM3 exhibited conserved amino acid sequences between the three species, such as the two transmembrane domains and the palmitoylation sites at positions 71, 72, and 105 (Fig. 7A), suggesting that swine IFITM3 may have posttranslational modification characteristics similar to the human counterpart. Thus, immunoprecipitation together with an acyl-biotin exchange (ABE) assay was performed to determine the S-palmitoylation of swine IFITM3. Western blotting results indicated that swine IFITM3 underwent S-palmitoylation in the Marc-145-IFITM3-flag cell line (Fig. 7B and C), while the palmitoylation modification was significantly weakened after cysteine was mutated to alanine and almost completely disappeared when all the three cysteines were mutated to alanine (Fig. 7D and E). To assess whether S-palmitoylation of IFITM3 was important for anti-PRRSV activity, single (C105A), double (CC71/72AA), and triple (CCC71/72/105AAA) cysteine to alanine mutant cell lines were infected with GFP-PRRSV for further analysis. Both Western blotting and FACS results showed that the single cysteine residue palmitoylation of IFITM3 was important for anti-PRRSV activity, as an almost complete loss of anti-PRRSV activity was observed when any of the cysteine residues were mutated (Fig. 7F and G). Then colocalization between mutant IFITM3 and endosomes or lysosomes was analyzed. Confocal results indicated that IFITM3-ΔPal (CCC71/72/105AAA) still largely colocalized with early/late endosomes and lysosomes in Marc-145-IFITM3-ΔPal-flag cells (Fig. 7M), consistent with the results of Fig. 4A. However, according to the Western blotting results of Fig. 7D and F, mutation of cysteine to alanine markedly decreased modified IFITM3 expression and abolished its antiviral effect, suggesting that S-palmitoylation of IFITM3 is critical for its anti-PRRSV activity.

Sequence alignment of IFITM3 showed that lysine residues are highly conserved between human, swine and monkey (Fig. 7A). Thus, whether swine IFITM3 underwent ubiquitin modification was determined. Compared with the Marc-145-Vector cells, IFITM3 ubiquitination was notably increased in Marc-145-IFITM3-flag cells (Fig. 7H and I). To further explore the specific sites where ubiquitination occurred, cell lines with individual lysine mutants as well as IFITM3 mutants in which three of the four lysines or all four lysines were mutated to alanine (ΔUb) were generated using a lentiviral transduction system. Western blotting after immunoprecipitation showed that all four lysine residues of swine IFITM3 were ubiquitinated (Fig. 7J), and almost complete loss of ubiquitinated bands could only be visualized when all four lysines were mutated to alanine (Fig. 7J). Subsequently, whether swine IFITM3 ubiquitination influenced its anti-PRRSV activity was investigated. Both Western blotting and FACS analysis of IFITM3 mutants deficient in ubiquitination for activity against PRRSV infection showed that mutation of any lysine to alanine resulted in IFITM3 almost completely losing its antiviral properties (Fig. 7K and L). Thus, whether ubiquitinated lysine influenced IFITM3 targeting to endosomes or lysosomes was determined. Indeed, IFITM3-ΔUb showed notably decreased colocalization with Rab5 and Rab7 compared with WT IFITM3 in Fig. 4A (Fig. 7M). Nevertheless most IFITM3-ΔUb still colocalized with lysosomes (Fig. 7M), suggesting that ubiquitination of IFITM3 might affect its anti-PRRSV activity by influencing its subcellular localization. Then the subcellular localization of PRRSV particles were determined. The results showed that PRRSV colocalized with early/late endosomes or lysosomes in both Marc-145-IFITM3-ΔPal-flag and Marc-145-IFITM3-ΔUb cells (Fig. 7N). Thus, in contrast to a previously reported role of IFITM3 posttranslational modifications on IAV (24), S-palmitoylation and ubiquitination of IFITM3 exhibited synergistic effects on regulation of its anti-PRRSV activity.

### IFITM3 is incorporated into PRRSV particles and diminishes viral infectivity.

**FIG 8:**
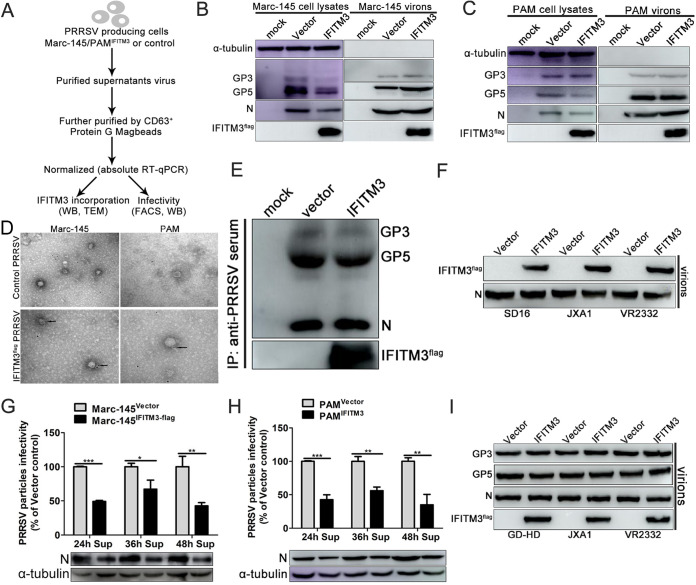
IFITM3 is a PRRS virion-associated protein. (A) Representation of the experimental scheme used. (B) Marc-145-Vector or Marc-145-IFITM3-flag cells were infected with 0.1 MOI PRRSV. At 48 hpi, both cell lysates and supernatants purified by ultracentrifugation through sucrose density gradient were collected and analyzed by Western blotting. (C) As described above, PAM lysates and purified virions were analyzed. (D) Virions produced as described above were analyzed using immunogold electron microscopy. Unfixed viral preparations produced from IFITM3-overexpressing or control cells were purified and incubated with anti-flag antibodies, followed by incubation with 6 nm gold-conjugated secondary antibody. Representative pictures are presented. Scale bar, 100 nm. (E) Viral supernatants produced from IFITM3-overexpressing or control cells were concentrated and purified, followed by immunoprecipitation using swine antiserum. Western blotting was performed using an anti-flag antibody to detect IFITM3 and using swine antiserum to detect PRRSV GP3 and GP5. (F) Virions of different PRRSV strains obtained as described above were subjected to Western blotting using anti-IFITM3 antibody, and N protein was used as the loading control. (G and H) Incorporation of IFITM3 into PRRS virions decreased viral infectivity. Newly produced virions from IFITM3-overexpressing or control cells were collected 24, 36, or 48 hpi, purified, and normalized prior to infectivity analysis. (I) IFITM3 did not affect PRRSV major envelope protein processing and incorporation into viral particles. Equal amounts of N protein were loaded into each lane, and Western blotting was performed using anti-flag, swine antiserum, and anti-N antibodies. PRRSV, porcine reproductive and respiratory syndrome virus; IFITM, interferon-induced transmembrane; MOI, multiplicity of infection; PAM, porcine alveolar macrophage; hpi, hours postinfection.

Studies have described an additional mechanism by which IFITMs are incorporated into newly produced HIV-1 particles that display reduced infectivity compared with their WT counterparts (25, 26). However, whether the incorporation of IFITM3 into virions was restricted to HIV or whether it applied to PRRSV was not established. To focus only on PRRSV intrinsic infectivity, all the subsequent assays were performed after purification and normalization of viral particles produced in IFITM3-overexpressing or control cells (Fig. 8A).

Cell lysates and viral preparations obtained from both Marc-145-IFITM3-flag and PAM-IFITM3-flag and corresponding control cells were analyzed using Western blotting. Under these conditions, the primary PRRSV envelop proteins GP3 and GP5 and the capsid protein N in all cell lysates exhibited robust expression (Fig. 8B and C). When purified and normalized virions were analyzed, there were no significant differences between the expression of GP3, GP5, and N protein in the preparations produced in the IFITM3-overexpressing or control cells (Fig. 8B and C). However, readily detectable quantities of IFITM3 were observed in viral fractions from both Marc-145-IFITM3-flag cells and PAM-IFITM3-flag (Fig. 8B and C), suggesting that IFITM3 could be a virion-associated protein. To further validate the finding that IFITM3 was a bona fide virion-associated protein, viruses produced from IFITM3-overexpressing or control cells were further subjected to immunogold electron microscopy using an antibody against the flag tag. Immunogold labeling results revealed that IFITM3 distributed at or closely to the virion envelope (Fig. 8D). Then, protein G MagBeads were coated with porcine antiserum and coincubated with supernatants collected from PRRSV-infected Marc-145-Vector or Marc-145-IFITM3-flag cells, and immunoprecipitation was performed. Western blotting results revealed that GP3, GP5, and N protein were readily detectable, and IFITM3 was detected simultaneously (Fig. 8E), further confirming the results shown in Fig. 8B and C. Next, the ability of IFITM3 to be incorporated in normalized purified viral preparations of different strains (GD-HD, JXA1, and VR2332) was assessed by Western blotting. In agreement with the above results, IFITM3 was readily detected in viral preparations from the other three PRRSV strains (Fig. 8F). These results indicate that IFITM3 is packaged in substantial quantities in PRRS virions.

Subsequently, the impact of IFITM3 on the infectivity of progeny virions was determined by infecting Marc-145 cells with normalized quantities of purified virions harvested at 24, 36, or 48 hpi, followed by determination of GFP-PRRSV positive cell percentage or N protein expression, which was indicative of virion infectivity. Notably, virus recovery from supernatants of Marc-145-IFITM3-flag cells revealed reduced virion infectivity compared with the WT at all three time points (Fig. 8G). To determine whether the infectivity deficit of PRRSV particles produced in the presence of IFITM3 was target cell type specific, normalized PRRS virions were used to challenge PAMs, and the percentage of GFP-PRRSV-positive PAMs or N protein expression was determined. Even using different target cells, the infectivity of viruses produced from PAM-IFITM3-flag also displayed a characteristic decrease (Fig. 8H), indicating that this phenomenon was independent of the target cell. Human IFITMs have been reported to impair HIV-1 infectivity by decreasing Env processing in the host cell and reducing virions’ Env incorporation (40). To determine the impact of IFITM3 on PRRSV envelope protein incorporation, GP3 and GP5 were determined in normalized purified virions from IFITM3-overexpressing or control cells of different strains. Notably, IFITM3 did not affect the level of viral envelope glycoproteins incorporated into virions of different PRRSV strains (Fig. 8I), which appeared to be inconsistent with the reported mode of action of IFITM3 on HIV (40).

### Endogenous IFITM3 is incorporated into PRRS virions, and knockdown of IFITM3 increases viral replication and infectivity.

**FIG 9:**
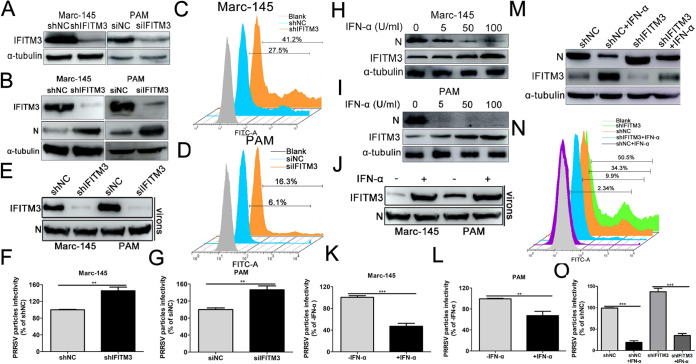
Endogenous IFITM3 is incorporated into PRRSV particles and reduces the virions’ intrinsic infectivity. (A) Western blotting of endogenous IFITM3 expression in Marc-145-shIFITM3 cells or PAMs transfected with 100 nM IFITM3-specific siRNA using an anti-IFITM3 antibody. (B to D) Marc-145-shIFITM3 cells or (B and D) PAMs transfected with IFITM3 siRNA were infected with 0.1 MOI GFP-PRRSV. At 36 hpi, cells were harvested for analysis of N protein expression using Western blotting or for analysis of GFP-PRRSV-positive cells using FACS. (E to G) Marc-145-shIFITM3 cells or PAMs transfected with IFITM3-specific siRNA were challenged with GFP-PRRSV at an MOI of 0.1 at 37°C for 1 h and subsequently incubated in 3% FBS+DMEM after extensive cell washing to remove input virus. At 36 hpi, newly produced virions were collected, purified, and normalized prior to Western blotting and infectivity analysis (expressed as GFP-PRRSV positive cells). (H and I) Western blotting of PRRSV infection in Marc-145 cells or PAMs pretreated with 0, 5, 50, or 100 U/ml IFN-α for 24 h. (J to L) Marc-145 cells or PAMs were treated with 100 U/ml IFN-α and then challenged with 0.1 MOI GFP-PRRSV. Virions retrieved from infected cell supernatants were purified and normalized for IFITM3 protein or infectivity assay. (M and N) Marc-145-shNC or Marc-145-shIFITM3 cells pretreated with 100 U/ml IFN-α for 24 h were infected with GFP-PRRSV at an MOI of 0.1. Cells were collected at 36 hpi for N protein or GFP-PRRSV-positive cell analysis. (O) Virions retrieved from the supernatants shown in panel M were purified and normalized for intrinsic infectivity detection. PRRSV, porcine reproductive and respiratory syndrome virus; IFN-α, interferon-α; IFITM, IFN-induced transmembrane; MOI, multiplicity of infection; PAM, porcine alveolar macrophage; siRNA, small interfering RNA; hpi, hours postinfection; NC, negative control.

Since overexpression of IFITM3 abrogated PRRSV replication (Fig. 1), we hypothesized that basal levels of IFITM3 possessed the potential to limit PRRSV replication as well. Marc-145 cells stably transfected with shIFITM3 or PAMs transfected with small interfering RNA (siRNA) targeting IFITM3 both significantly reduced endogenous IFITM3 expression (Fig. 9A). Western blotting results revealed that knockdown expression of endogenous IFITM3 significantly enhanced PRRSV infection in both Marc-145 cells and PAMs (Fig. 9B), and FACS analysis also showed similar results (Fig. 9C and D), further confirming that basal levels of IFITM3 were effective as an anti-PRRSV factor.

To further assess the role of endogenous IFITM3 in PRRSV infection, Marc-145-shIFITM3 cells or PAMs transfected with IFITM3 siRNA were challenged with 0.1 MOI of GFP-PRRSV, and newly produced virions were then purified and normalized, and their infectivity was assessed (according to the scheme provided in Fig. 8A). Intracellular reduction of IFITM3 led to a small concomitant reduction of virion-associated IFITM3 when sufficient N protein was loaded (Fig. 9E). When newly produced virions were normalized and used to challenge Marc-145 cells or PAMs, the infectivity of virions produced in IFITM3-knockdown cells significantly increased compared with the control cells (Fig. 9F and G). Thus, depletion of endogenous IFITM3 resulted in an increase in the intrinsic infectivity of progeny PRRS virions, corroborating the results presented in Fig. 8G and H.

Given that IFITM3 is an ISG, the role of IFITM3 in mediating the anti-PRRSV activity of IFN-α was investigated. Marc-145 cells or PAMs were stimulated for 24 h with IFN-α prior to infection with GFP-PRRSV. Western blotting results suggested that the abundance of IFITM3 protein in both Marc-145 cells and PAMs increased markedly in an IFN-α dose-dependent manner (Fig. 9H and I), suggesting that expression of IFITM3 was induced by IFN-α in both PRRSV permissive cells. In contrast to the upward trend of IFITM3, expression of PRRSV N protein decreased gradually with the increase in IFN-α (Fig. 9H and I). Additionally, following IFN-α stimulation, IFITM3 incorporation of newly produced virions was readily detectable (Fig. 9J). When virions produced under these conditions were purified, normalized, and used to challenge Marc-145 cells or PAMs, a significant decrease in infectivity was observed from virions produced in IFN-α-treated cells versus viruses produced in the control cells (Fig. 9K and L).

To verify whether IFITM3 mediated the anti-PRRSV activity of IFN-α, Marc-145-shNC/-shIFITM3 cells treated with IFN-α were infected with GFP-PRRSV and then analyzed via Western blotting and FACS. Western blotting results showed that the N protein level in IFN-α-treated shIFITM3 cells was significantly increased compared with the shNC cells treated with IFN-α (Fig. 9M), and FACS analysis showed similar results (Fig. 9N), suggesting that IFITM3 partially mediated the anti-PRRSV activity of IFN-α. In the following conditions, purified and normalized virions were inoculated to Marc-145 cells to evaluate their infectivity. Virions from IFN-α-treated Marc-145-shNC cells showed dramatically decreased infectivity compared with virions from Marc-145-shNC cells (Fig. 9O), while knockdown of IFITM3 expression partially increased the virions’ infectivity compared with shNC cells treated with IFN-α (Fig. 9O). Together, these results suggest that endogenous IFITM3 is incorporated into PRRSV particles and positively interferes with their infectivity.

### IFITM3 effectively blocks PRRSV cell-to-cell transmission.

**FIG 10:**
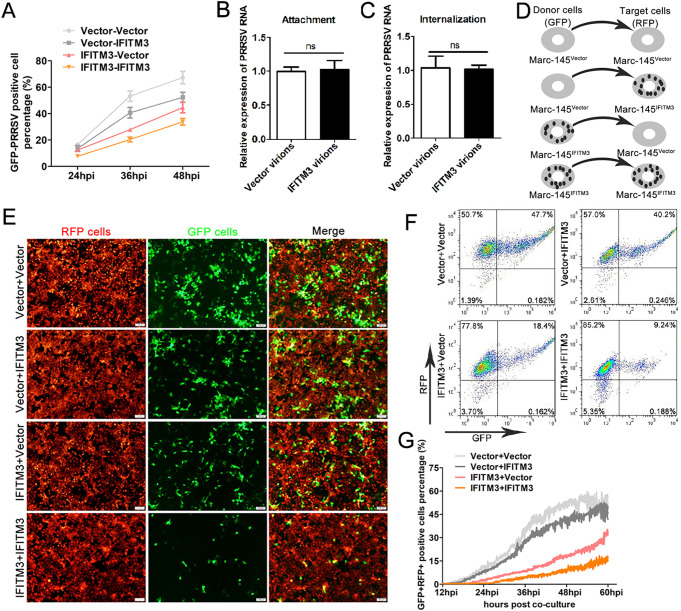
IFITM3 in virus-producing cells restricts PRRSV cell-to-cell transmission. (A) GFP-PRRSV particles retrieved from Marc-145-Vector or Marc-145-IFITM3-flag cells were purified, normalized, and then infected with Marc-145-Vector or Marc-145-IFITM3-flag cells. At 24, 36, or 48 hpi, cells were collected for GFP-PRRSV-positive cell analysis using FACS. (B) PRRSV particles from Marc-145-Vector or Marc-145-IFITM3-flag cells were purified and normalized, and Marc-145 cells were challenged with the viral particles for 1 h on ice. Unabsorbed virus was washed away using ice-cold PBS, and cells were harvested for PRRSV genome analysis using RT-qPCR. In another parallel group, cells were incubated with 37°C prewarmed DMEM and shifted to 37°C for 1 h after washing using PBS. After trypsin treatment and washes with PBS, cells were used for PRRSV genome detection via RT-qPCR (C). Marc-145-Vector cells were infected with GFP-PRRSV (0.1 MOI). When ∼60% of the cells were GFP positive, cells were sorted and used as donor cells to coculture with Marc-145-IFITM3-flag-RFP cells (red, target cells) for 2 h. Target cells were sorted and cultured, and transmission was monitored by the fraction of GFP+RFP+ target cells at the indicated time points. (D) Schematic illustration of the four combinations of coculture systems. (E) Representative images of coculture cell viral transmission experiments were extracted 48 hpi. (F) Flow cytometry analysis of GFP+RFP+ double positive cells in the various coculture systems, 48 hpi. (G) Real-time time-lapse microscopy analysis of GFP-PRRSV intercellular spread. Marc-145-Vector-RFP or Marc-145-IFITM3-flag-RFP cells were infected with GFP-PRRSV at an MOI of 0.1. When ∼60% of the cells were GFP-PRRSV positive, the cells were digested, sorted, and counted as donor cells to coculture with target cells to produce four coculture conditions. Images were taken every 5 min for 60 hpi. The area of GFP+RFP+ pixels was quantified and used as a marker of target cell infection. A total of three fields of view in two independent experiments were analyzed and plotted as the mean ± the standard error of the mean; one representative experiment is shown. PRRSV, porcine reproductive and respiratory syndrome virus; IFITM, interferon-induced transmembrane; MOI, multiplicity of infection; RT-qPCR, reverse transcription-quantitative PCR; hpi, hours postinfection.

Since virions from virus-producing cells in the presence of IFITM3 exhibited decreased infectivity, we evaluated the antiviral effects of IFITM3 in different infection modes. Purified and normalized virions from IFITM3-overexpressing or control cells were inoculated to Marc-145-Vector or Marc-145-IFITM3-flag cells, and then the GFP-PRRSV positive cell percentage under different infection modes was analyzed using FACS at different time points. Virions from Marc-145-Vector cells showed stronger infective activity when inoculated with either Marc-145-Vector or Marc-145-IFITM3-flag cells (Fig. 10A). Virions in the presence of IFITM3 resulted in the infection of fewer target cells overall compared with virions in the absence of IFITM3 (Fig. 10A), and a more potent inhibition was observed when both virions and target cells contained IFITM3 (Fig. 10A). Thus, whether IFITM3 impeded newly assembled IFITM3-containing virion attachment or entry into host cells was determined. Purified and normalized virions were inoculated to Marc-145 cells, and viral attachment and an internalization assay were performed by evaluating viral genome content. However, virions from IFITM3-overexpressing or control cells did not exhibit any differences in the ability of adsorption or entry into host cells (Fig. 10B and C). A previous study showed that IFITM3 incorporated into HIV virions could effectively impair virion spread in target cells (26), and for PRRSV, intercellular transmission was an important means for establishment of sustained and effective infection (41). This led us to ask whether IFITM3 exhibited its anti-PRRSV function by impairing intercellular virion spread.

To determine whether IFITM3 could restrain PRRSV intercellular spread during infection, we performed a dynamic analysis of PRRSV spread using a coculture system. GFP-PRRSV-positive cells sorted by flow cytometry were used as the donor cells in different coculture systems, whereas cells stably expressing a red fluorescent protein (RFP) marker (tomato red) were utilized as the target cells; the various combinations are illustrated in Fig. 10D. For the different coculture systems, target cells were plated in a 35-mm glass-bottom petri dish and visualized by time-lapse fluorescence microscopy with images acquired every 5 min for up to 60 hpi. Representative videos of PRRSV intercellular transmission in different coculture modes are presented in Movies S1 and S2. Live cell time-lapse analysis results showed that in the absence of IFITM3 in donor cells, PRRS virions efficiently spread to target cells, as shown by the presence of GFP+RFP+ cells, and eventually formed cluster-like cell clumps (Fig. 10E, upper panel and Fig. 10F), and a slight decrease (0.157-fold) of GFP+RFP+ double positive cells was observed when Marc-145-IFITM3-flag cells were utilized as target cells versus Marc-145-Vector cells as the target cells 48 hpi (Fig. 10E and F). In addition, the presence of IFITM3 in the donor cells further abolished the spread of virions, as a 0.77-fold decrease in the number of GFP+RFP+ Marc-145-IFITM3-flag target cells was observed at 48 hpi (Fig. 10F). Under these experimental conditions, the strongest inhibitory effect on PRRSV intercellular transmission was observed in the presence of IFITM3 in both donor and target cells (Fig. 10E and F). Real-time time-lapse microscopy analysis of PRRSV spread also showed that IFITM3 could effectively hamper PRRSV intercellular transmission (Fig. 10G).

## DISCUSSION

Several studies have extensively explored the transport process of viruses following entry into host cells, and the endosome-lysosome pathway, although not the exclusive entry mechanism, is considered to be a classic early intracellular transport process. Receptor-mediated PRRSV entry into cells occurs through adsorption and endocytosis; however, its intracellular transport process is still unclear. Previous studies have suggested that PRRSV particles are trafficked through early endosomes, but not late endosomes or lysosomes, for productive infection (37). In the present study, it was shown that, similar to several other enveloped viruses, PRRSV particles were transported to early/late endosomes and lysosomes and then underwent membrane fusion to release genomic RNA and initiate their replication process. Since subcellular localization is coincident for both Marc-145 cells and PAMs, our results are likely to objectively reflect the intracellular transport process of PRRSV following entry into host cells. Although no significant differences were observed in restriction of PRRSV attachment and entry after overexpression of IFITM3, taking into consideration the PRRSV replication kinetics in IFITM3-overexpressing or control cells and the localization of IFITM3 at both endosomes and lysosomes, we speculate that IFITM3 is following the established paradigm of acting at the later stages of entry of PRRSV. Colocalization of IFITM3 with PRRSV particles and endosomes or lysosomes showed that PRRSV particles are likely cotrafficked with IFITM3-containing endosomes (Fig. 4), which is consistent with previous reports showing that the IFITM3-sensitive virus IAV was cotrafficked with IFITM3-positive endosomes during intracellular transportation (42). Virus restriction by IFITM3 was mediated partially by its ability to prevent virus-endosomal membrane fusion (also known as lipid mixing) (42). Furthermore, IFITM3-induced cholesterol accumulation in cellular compartments blocks virus entry mediated by lipid mixing (23), although a previous study also documented unimpeded IAV lipid mixing activity in IFITM3-expressing cells (38). According to the results of the present study, overexpression of IFITM3 resulted in notable accumulation of cholesterol in endosomes and lysosomes, and blockage of cholesterol efflux effectively impaired PRRSV membrane fusion (Fig. 6). Analysis of lipid dye dequenching showed significant differences in the rate of lipid mixing in IFITM3-overexpressing or control cells, in agreement with reduced IAV lipid dequenching in IFITM3-expressing cells (43). Thus, we postulate that PRRS virions are trapped in endosomes or lysosomes following endocytosis due to IFITM3-induced alterations of cholesterol at endosomes and lysosomes, which prevents viral fusion and, subsequently, genomic release into the host cell’s cytoplasm.

Posttranslational modifications of IFITM3 have been shown to serve a crucial role in subcellular localization and antiviral function. S-palmitoylation of Cys-72 of human IFITM3 is essential for restriction of IAV and DENV (44, 45). The addition of palmitoyl groups is associated with IFITM3 stability, since mutation of any of the cysteines to alanine resulted in a notable decrease in total IFITM3 expression and almost completely abolished the anti-PRRSV activity of IFITM3, thus explaining the importance of S-palmitoylation for the antiviral activity of the IFITM3. Interestingly, no changes were observed in IFITM3 subcellular localization when all cysteine residues were mutated, suggesting that S-palmitoylation of swine IFITM3 primarily maintains protein stability without affecting its subcellular localization. A previous study showed that S-palmitoylation and ubiquitination served different roles in suppressing IAV replication (24). The present discovery of IFITM3 ubiquitination and analysis of a ubiquitination-deficient mutant on PRRSV infection showed that ubiquitination is a necessary posttranslational modification of IFITM3 and is essential for effective inhibition of PRRSV infection. Complete mutation of lysine to alanine of IFITM3 results in reduced localization with endosomal markers but not lysosomal markers (Fig. 7M); considering the almost complete abrogation of its antiviral activity, we speculate that appropriate subcellular localization of IFITM3 is necessary for its anti-PRRSV activity. In disagreement with a previous study (24), the effects of IFITM3 posttranslational modification on IAV replication, S-palmitoylation, and ubiquitination served a synergistic role in inhibiting PRRSV replication via different molecular mechanisms in the present study.

The results of the present study highlight IFITM3 as an anti-PRRSV factor that acts primarily through the sequestration of infective virions in endosomes or lysosomes. On the other hand, an additional mechanism of viral interference with which IFITMs could lead to the formation of virions with decreased infectivity has been previously reported in HIV and some other viruses (25, 26, 46, 47). By utilizing overexpression or induction of IFITM3 in PRRSV permissive cells, we showed for the first time that IFITM3 was a PRRSV-associated host protein that can diminish intrinsic infectivity of progeny viral particles through wrapping as part of the virus envelope. Furthermore, knockdown of IFITM3 expression affected viral sensitivity to IFN-α, suggesting the important role of IFITM3 in mediating the antiviral activity of IFN-α. Previous reports suggested that incorporation of IFITM3 into HIV virions resulted in impaired envelope protein Env processing and incorporation into HIV-1 virions (40); however, no differential effects of swine IFITM3 on PRRSV envelop protein GP3 and GP5 processing and incorporation in PRRS virions was observed in the present study. IFITMs have been proposed to sterically rigidify membranes in which they are inserted, leading to fusion inhibition (38). The finding that IFITMs can interact with themselves strongly supports the above hypothesis, although the extent of this multimerization has not been fully examined (48). Whether these mechanisms of inhibition are at play in target cells and in virion particles remains to be determined. The present study expands our understanding of how IFITM proteins contribute to intrinsic immunity, and it could prove fruitful to study these functions in the context of other enveloped arteriviruses, which may reveal whether viruses sensitive to IFITM3 in the target cell are also susceptible to this donor cell-specific mode of antiviral function, that is, virion incorporation and decreased infectivity.

Nevertheless, it is still unclear whether the physical incorporation of IFITM3 into PRRSV particles is indispensable for effective anti-PRRSV activity or whether the above-described two properties can be dissociated. However, we are more inclined to the view that IFITM3 exerts it antiviral effect through two different molecular mechanisms, since viral membrane fusion, which is essential for infection, was blocked during one viral replication cycle when WT PRRSV was utilized, and this process does not appear to be associated with viral assembly or production of new virions. Additionally, the most conspicuous anti-PRRSV effect was observed when IFITM3 was present in both target and donor cells. In this infection mode, although no marked difference was observed in absorption or entry capability of newly produced virions from IFITM3-overexpressing or control cells, it is very likely that the reduced virion infectivity as well as the membrane fusion ability blocked by the presence of IFITM3 after entering the endosomes/lysosomes both served key roles. Identification of IFITM3 mutants that have lost their ability to be incorporated into viral particles may help us validate the above hypothesis. Given that the protection offered by antiviral factors is often incomplete, this dual mechanism of inhibition is likely to potentiate the leverage of IFITM3 on PRRSV replication.

## MATERIALS AND METHODS

### Cells, viruses, and reagents.

PAMs were prepared from 4- to 6-week-old PRRSV-negative pigs as previously described (49). PAMs were maintained in RPMI 1640 medium (Gibco, Thermo Fisher Scientific, Inc.) supplemented with 10% fetal bovine serum (FBS) (Biological Industries), 100 U/ml penicillin, and 100 μg/ml streptomycin. Marc-145 and HEK293T cells were cultured in DMEM (Gibco, Thermo Fisher Scientific, Inc.) supplemented with 10% FBS. All cells were cultured at 37°C with humidity and 5% CO_2_. The animal experiments were performed in strict accordance with the guidelines of the Institutional Animal Care and Use Committee and approved by the Animal Care and Use Committee of Henan Agricultural University Zhengzhou (Henan, China).

Two highly pathogenic PRRSV strains, SD16 (GenBank accession no. JX087437.1) and JXA1 (GenBank accession no. EF112445.1), and PRRSV-2 prototype strain VR-2332 (GenBank accession no. EF536003.1) were used in the present study. Enhanced green fluorescent protein (EGFP)-PRRSV based on the genetic background of the SD16 strain that expressed EGFP as an additional open reading frame (ORF) (rHP-PRRSV/SD16/EGFP) was kindly provided by En-min Zhou at Northwest A&F University (50). Viruses were propagated and titrated in Marc-145 cells by calculating the median tissue culture infective dose as previously described (33). SD16 PRRSV was used for the majority of the experiments, and thus is indicated as PRRSV unless otherwise specified.

The lipophilic fluorescent probe 3,3′-dioctadecyloxacarbocyanine (DiOC18) and octadecyl rhodamine B (R18) were both purchased from Invitrogen (Thermo Fisher Scientific, Inc.). Cholesterol flow specific inhibitor U18666A, endosomal acidification inhibitor bafilomycin A1 (BafA1), and amphotericin B (AmphoB) were all obtained from MedChemExpress. Mouse anti-α-tubulin/-flag monoclonal antibody was obtained from Sigma-Aldrich, Merck KGaA, and rabbit anti-IFITM3 polyclonal antibody was obtained from ProteinTech Group, Inc. Rabbit anti-Rab5 (early endosome marker), -M6PR (late endosome marker), and -LAMP2 (lysosome marker) antibodies were obtained from Abcam. Rabbit anti-flag monoclonal antibody was purchased from the Beyotime Institute of Biotechnology. Colloidal gold AffiniPure goat anti-rabbit IgG (H&L) and DyLight 405-AffiniPure goat anti-rabbit IgG (H&L) were purchased from Jackson ImmunoResearch Laboratories, Inc. The cholesterol staining reagent, Filipin, was purchased from Sigma-Aldrich, Merck KGaA. Alexa Fluor 488-conjugated goat anti-rabbit IgG (H&L) and Alexa Fluor 594-conjugated goat anti-mouse IgG (H&L) were obtained from Abcam.

### Overexpression and knockdown of IFITM3 in PRRSV permissive cells.

Swine IFITM3 cDNA with an N-terminal flag tag was cloned into pTRIP-puro lentiviral expression vector to generate pTRIP-IFITM3-flag. The recombinant plasmid was verified by sequencing. A blank pTRIP-puro vector was generally used as a control. pTRIP-IFITM3-flag expression vector (1.3 μg) was cotransfected into HEK293T cells, with packaging vectors psPAX (1.8 μg) and pMDG.2 (1.0 μg) to produce pseudotyped lentiviral vectors using X-tremeGENE HP DNA transfection reagent (Roche Diagnostics). Supernatants containing the pseudotyped lentiviruses were collected 48 h posttransfection, followed by centrifuging at 1,000 rpm for 5 min at 4°C to remove cell debris.

To create cell lines stably expressing IFITM3, Marc-145 cells were infected with pseudovirions in the presence of 1 μg/ml Polybrene (Sigma-Aldrich, Merck KGaA). A total of 48 h after infection, cells were selected in 10% FBS + Dulbecco modified Eagle medium (DMEM) containing 8 μg/ml puromycin (Sigma-Aldrich, Merck KGaA). For the production of red fluorescence cell lines stably expressing IFITM3, Marc-145-IFITM3-flag cells were first inoculated with pseudovirions expressing red fluorescence (packaged using pLenti-tomato-neo expression vector together with psPAX and pMDG.2) and further screened using 600 mg/ml neomycin, and the control cell lines were established as described above. Marc-145 cell lines with different mutant versions of IFITM3 (Cys→Ala, CC71/72AA, C105A, CCC71/72/105AAA; Lys→Ala, K24A, K83A, K88A, K104A, K(24)A, K(83)A, K(88)A, K(104)A, KKKK24/83/88/104AAAA) were constructed by cloning mutant genes into pTRIP-puro using overlap PCR with mutant primers, and all cell lines were selected using puromycin screening and identified by indirect immunofluorescence assay (IFA) and Western blotting. The sequences of the primers used in the present study are listed in Table 1.

**TABLE 1:** Primers used in this study

Primer name	Sequence (5′–3′)
IFITM3-qF	GTCGTCTGGTCCCTGTTCAAC
IFITM3-qR	GAGTAGGCGAAAGCCACGAA
IFITM3-F (PCR)	CCCTCGAGATGGATTACAAGGATGACGACGATAAGATGAACTGCGCTTCCCAGCC
IFITM3-R (PCR)	CGGGATCCCTAGTAGCCTCTGTAATCCTTTATGAGCTGCAGAACT
IFITM3 CC71/72AA-F	CTCTTCATGAACTGGGCTGCTCTGGGCTTCGTGGCTTTCG
IFITM3 CC71/72AA-R	CGAAAGCCACGAAGCCCAGAGCAGCCCAGTTCATGAAGAGG
IFITM3 C105A-F	TCCACCGCCAAGGCTCTGAACATCTGGGCTC
IFITM3 C105A-R	GAGCCCAGATGTTCAGAGCCTTGGCGGTGGA
IFITM3 K24A-F	TATGAGATGCTCGCTGAGGAGCACGAGGTGGC
IFITM3 K24A-R	GCCACCTCGTGCTCCTCAGCGAGCATCTCATA
IFITM3 K83A-F	CGCCTACTCCGTGGCTGCGAGGGACCGGAAGATGG
IFITM3 K83A-R	CTTCCGGTCCCTCGCAGCCACGGAGTAGGCG
IFITM3 K88A-F	GGCGAGGGACCGGGCTATGGTGGGAGACA
IFITM3 K88A-R	TGTCTCCCACCATAGCCCGGTCCCTCGCCTTCACG
IFITM3 K104A-F	GAGCTATGCCTCCACCGCCGCTTGCCTGAAC
IFITM3 K104A-R	GTTCAGGCAAGCGGCGGTGGAGGCATAGCTC

To generate Marc-145 cell lines with IFITM3 expression knocked down, Marc-145 cells were infected with recombinant pseudovirions and selected in 10% FBS+DMEM containing 8 μg/ml puromycin. Lentiviral particles were generated via transfection of HEK293T cells with psPAX, pMDG.2, and pLKO.1-puro constructs expressing short hairpin RNA (shRNA) against IFITM3 or empty vector control expressing a stuffer sequence. The knockdown efficiency was verified by Western blotting. The sequences of the shRNA targeting monkey IFITM3 and control shRNA were shRNA were: shRNA-IFITM3, 5′-CCGGGCAGCTCATAAAGGATTACTTCTCGAGAAGTAATCCTTTATGAGCTGCTTTTTG-3′ and shRNA-control, 5′-CCGGGCACTACCAGAGCTAACTCACTCGAGTGAGTTAGCTCTGGTAGTGCTTTTTG-3′. To knock down endogenous IFITM3 expression in PAMs, a pair of small interfering RNAs (siRNAs) targeting swine IFITM3 was synthesized; the sequences were: sense, 5′-UUCUCCGAACGUGUCACGUTT-3′ and antisense, 5′-ACGUGACACGUUCGGAGATT-3′. IFITM3 knockdown efficiency was determined via Western blotting.

**Purification of PRRS virions.** Marc-145-Vector or Marc-145-IFITM3-flag cells were infected with PRRSV at a multiplicity of infection (MOI) of 0.1, and supernatants were harvested 48 h postinfection (hpi) and purified by centrifugation (4,000 × *g*, 4°C, 30 min). The supernatant was concentrated by ultracentrifugation (40,000 rpm, 3 h, 4°C; Beckman Coulter). The concentrated virus was then resuspended in phosphate-buffered saline (PBS; pH 7.4) and purified over a 30 to 60% sucrose gradient (40,000 rpm, 3 h, 4°C), and the banded virus was collected, diluted with PBS, pelleted (40,000 rpm, 3 h, 4°C), and resuspended in 1 ml PBS for further use. Taking into consideration that IFITM3 was reported to be associated with exosomes and given that exosomes may share numerous characteristics with virion particles (51), anti-CD63 immuno-magnetic beads (Invitrogen, Thermo Fisher Scientific, Inc.) were used to exclude free exosome contamination and further purify virions, according to the manufacturer’s protocol.

### Immunogold labeling of purified virions for transmission electron microscopy.

Transmission electron microscopy was performed as previously described (52) with the following modifications: purified PRRSV particles were diluted 1:20 with PBS and adsorbed onto carbon-coated 200-mesh copper palladium grids. After washing with PBS for 5 min, the samples were blocked with 3% BSA in PBS for 1 h at room temperature (RT). Mouse anti-flag tag monoclonal antibody (Beyotime Institute of Biotechnology) was diluted in 1% BSA/PBS (1:500) and adsorbed to the grid for 1 h at RT. Following 3 washes with PBS, a goat anti-mouse IgG conjugated with 6-nm gold particles (Jackson ImmunoResearch Laboratories, Inc.) was added and incubated at RT for 1 h. After labeling, the grids were washed 3 times using PBS, and then the grids were negatively stained with aqueous 3% uranyl acetate solution for 15 sec. Images of stained virions were captured on a Hitachi H-7650 120 kV transmission electron microscope.

### Cell-to-cell transmission assay.

A cell coculture system was utilized to assess intercellular viral spread. Marc-145-Vector or Marc-145-IFITM3-flag cells were inoculated with 0.1 MOI GFP-PRRSV for 48 h to achieve ∼60% GFP-PRRSV-positive cells. Cells were adequately digested with trypsin to ensure that a single cell suspension was obtained, and then the GFP-PRRSV-positive cells were separated using a FACSAria cell sorter and were used as the donor cells after counting using trypan blue staining. For the virus cell-to-cell transmission assay utilizing Marc-145-Vector-RFP cells as the target cells, 5 × 10^5^ donor cells were mixed with 5 × 10^5^ Marc-145-Vector-RFP target cells (∼1:1 donor:target) in 6-well plates. After coculturing for 2 h at 37°C, cells were adequately digested, and ∼4 × 10^5^ target cells (red) were separated from donors by flow cytometry sorting and plated in 35-mm culture dishes and continued to incubate for 12 h at 37°C. Then time-lapse video imaging was performed. Transmission and fluorescence images were taken every 5 min for up to 60 hpi at 37°C with 5% CO_2_, using a Nikon Biostation IMQ. At 48 hpi, another batch of parallel samples was subjected to flow cytometry detection, and data were analyzed using FlowJo version 7.6 (FlowJo LLC). When Marc-145-IFITM3-flag-RFP cells were used as the donor cells, the other two coculture systems were similar to those described above.

### Virion double fluorescence labeling and single virion imaging and analysis.

PRRSV particles’ lipid envelopes were labeled using DiOC18 (Thermo Fisher Scientific, Inc.) and R18 (Thermo Fisher Scientific, Inc.) as previously described (53, 54) with some modifications. Purified PRRSV particles were resuspended in 1 ml PBS, followed by addition of DiOC18 and R18 to final concentrations of 0.2 and 0.4 μM, respectively. The mixture was agitated vigorously and incubated in the dark for 1 h at RT and subsequently filtered through a 0.22-μm-pore size filter (EMD Millipore) to remove any large lipid and/or virus aggregates. The labeled virions were purified from excess dyes through a NAP-5 gel filtration column (GE Healthcare) that was equilibrated with 50 mM HEPES (pH 7.4) and 145 mM NaCl solution. The virions were aliquoted into tubes and stored at −80°C until use.

For single virus imaging, Marc-145 cells were seeded onto 35-mm collagen-coated glass-bottom petri dishes and were prechilled on ice for 30 min and then incubated with labeled virus on ice for 30 min to allow virus to fully adsorb to the cell surface. The cells were washed 3 times using ice-cold PBS to remove any unbound viruses. Viral entry was initiated by adding 2 ml of prewarmed (37°C) 3% FBS+DMEM, and then the cells were imaged immediately on the stage of a confocal microscope. Two ranges of fluorescence wavelength, 510 to 525 nm and 575 to 640 nm, were detected simultaneously. Every 15 sec, three Z-stacks separated by ∼2 μm were acquired to cover the thickness of cells using a Zeiss 63x/1.4NA oil lens objective. For the viral membrane fusion experiments, a DiOC18 dequenching (fusion) curve for the individual virion was acquired by tracking particles using the R18 (red) channel and the DiOC18 (green) channel. The fusion events were manually obtained from fluorescence intensity that showed at least 2-fold increases in DiOC18 fluorescence. The dequenching time (fusion time) was calculated as the time taken to reach an intensity plateau from the time of initial rise in intensity of DiOC18. To block PRRSV fusion, experiments were performed in medium supplemented with 70 mM NH_4_Cl (pH 7.6) or containing 200 nM of the endosomal acidification inhibitor BafA1. For quantitative analysis, PRRSV particle total fluorescence intensities were analyzed using ImageJ.

### Colocalization analysis.

Marc-145 cells or PAMs were seeded on coverslips in 24-well plates at a density of 1 × 10^4^ cells/well 1 day prior to prechilling on ice for 30 min, followed by infecting with 0.1 MOI of PRRSV and further incubation on ice for 30 min. At 10, 30, and 60 min postinfection, cells were fixed with 4% paraformaldehyde for 10 min at RT, permeabilized with 0.1% Triton X-100 for 30 min, and blocked with 2% BSA for 1 h at RT. Cells were stained using mouse anti-PRRSV N antibody (1:300; produced in our lab) together with rabbit anti-Rab5 (1:1,000; ProteinTech Group, Inc.), -M6PR (1:1,000; ProteinTech Group, Inc.), or -LAMP2 antibody (1:1,000; ProteinTech Group, Inc.) for 1 h at RT. Then, cells were incubated with Alexa Fluor 594-conjugated goat anti-mouse IgG (H&L) and Alexa Fluor 488-conjugated goat anti-rabbit IgG (H&L) (1:500; Abcam) for 1 h at RT in the dark. Coverslips were mounted on glass slides with DAPI counterstain (Sigma-Aldrich, Merck KGaA). Coverslips were imaged using a Zeiss LSM510 laser scanning inverted confocal microscope with a Zeiss 63x/1.4NA oil lens objective.

For subcellular distribution of endosomes, lysosomes, IFITM3, and cholesterol, Marc-145-IFITM3-flag cells transfected with pcDNA3.1-Rab5-GFP, pcDNA3.1-Rab7, or pcDNA3.1-LAMP1-YFP were stained with mouse anti-flag, rabbit anti-Rab7 (1:500; Beyotime Institute of Biotechnology) antibodies and Alexa Fluor 594-conjugated goat anti-mouse IgG (H&L) or Alexa Fluor 488-conjugated goat anti-rabbit IgG (H&L) (1:500; Abcam). Cellular distribution of cholesterol was examined by incubation with 50 μg/ml filipin (Sigma-Aldrich, Merck KGaA) for 2 h at RT in the dark after incubation with the secondary antibodies. Image analysis to determine colocalization was performed using ImageJ.

### Measurement of viral particle infectivity.

Viral infectivity assays were performed using purified, normalized viral preparations. Prior to the viral infectivity assay, a plasmid containing a 372-bp fragment of the PRRSV ORF7 sequence was utilized to create a standard curve to conduct absolute reverse transcription-quantitative PCR (RT-qPCR) to ascertain supernatant virus copies for PRRS virion normalization. To approximate a single cycle of PRRSV infection and facilitate detection, Marc-145 cells were inoculated with an MOI of 1.0 of purified and normalized GFP-PRRSV from supernatants of cells of the indicated treatment, and the percentage of infected cells was monitored at one viral replication cycle (∼10 h) by FACS to avoid multiple cycles of infection. Viral infection activity was expressed as the normalized proportion of GFP-PRRSV positive cells.

### Statistical analysis.

Statistical analysis was performed using GraphPad Prism (GraphPad Software, Inc.). Data are presented as the mean ± standard deviation. Differences between two groups were assessed using an unpaired Student’s *t* test, while a one-way analysis of variance (ANOVA) was used to compare differences between three or more groups. *P* < 0.05 was considered to indicate a statistically significant difference. All experiments were performed in triplicate.

## Supplementary Material

Supplemental file 1

Supplemental file 2

Supplemental file 3

## References

[1] Schneider WM, Chevillotte MD, Rice CM. 2014. Interferon-stimulated genes: a complex web of host defenses. Annu Rev Immunol 32:513–545. doi:10.1146/annurev-immunol-032713-120231. 24555472 PMC4313732

[2] Schoggins JW. 2014. Interferon-stimulated genes: roles in viral pathogenesis. Curr Opin Virol 6:40–46. doi:10.1016/j.coviro.2014.03.006. 24713352 PMC4077717

[3] Smith SE, Weston S, Kellam P, Marsh M. 2014. IFITM proteins: cellular inhibitors of viral entry. Curr Opin Virol 4:71–77. doi:10.1016/j.coviro.2013.11.004. 24480526 PMC7185728

[4] Diamond MS, Farzan M. 2013. The broad-spectrum antiviral functions of IFIT and IFITM proteins. Nat Rev Immunol 13:46–57. doi:10.1038/nri3344. 23237964 PMC3773942

[5] Perreira JM, Chin CR, Feeley EM, Brass AL. 2013. IFITMs restrict the replication of multiple pathogenic viruses. J Mol Biol 425:4937–4955. doi:10.1016/j.jmb.2013.09.024. 24076421 PMC4121887

[6] Narayana SK, Helbig KJ, McCartney EM, Eyre NS, Bull RA, Eltahla A, Lloyd AR, Beard MR. 2015. The interferon-induced transmembrane proteins, IFITM1, IFITM2, and IFITM3 inhibit hepatitis C virus entry. J Biol Chem 290:25946–25959. doi:10.1074/jbc.M115.657346. 26354436 PMC4646249

[7] Brass AL, Huang IC, Benita Y, John SP, Krishnan MN, Feeley EM, Ryan BJ, Weyer JL, van der Weyden L, Fikrig E, Adams DJ, Xavier RJ, Farzan M, Elledge SJ. 2009. The IFITM proteins mediate cellular resistance to influenza A H1N1 virus, West Nile virus, and dengue virus. Cell 139:1243–1254. doi:10.1016/j.cell.2009.12.017. 20064371 PMC2824905

[8] Feeley EM, Sims JS, John SP, Chin CR, Pertel T, Chen LM, Gaiha GD, Ryan BJ, Donis RO, Elledge SJ, Brass AL. 2011. IFITM3 inhibits influenza A virus infection by preventing cytosolic entry. PLoS Pathog 7:e1002337. doi:10.1371/journal.ppat.1002337. 22046135 PMC3203188

[9] Weidner JM, Jiang D, Pan XB, Chang J, Block TM, Guo JT. 2010. Interferon-induced cell membrane proteins, IFITM3 and tetherin, inhibit vesicular stomatitis virus infection via distinct mechanisms. J Virol 84:12646–12657. doi:10.1128/JVI.01328-10. 20943977 PMC3004348

[10] Bailey CC, Huang IC, Kam C, Farzan M. 2012. Ifitm3 limits the severity of acute influenza in mice. PLoS Pathog 8:e1002909. doi:10.1371/journal.ppat.1002909. 22969429 PMC3435252

[11] Kenney AD, McMichael TM, Imas A, Chesarino NM, Zhang LZ, Dorn LE, Wu Q, Alfaour O, Amari F, Chen M, Zani A, Chemudupati M, Accornero F, Coppola V, Rajaram MVS, Yount JS. 2019. IFITM3 protects the heart during influenza virus infection. Proc Natl Acad Sci U S A 116:18607–18612. doi:10.1073/pnas.1900784116. 31451661 PMC6744864

[12] Ranjbar S, Haridas V, Jasenosky LD, Falvo JV, Goldfeld AE. 2015. A role for IFITM proteins in restriction of Mycobacterium tuberculosis infection. Cell Rep 13:874–883. doi:10.1016/j.celrep.2015.09.048. 26565900 PMC4916766

[13] Munoz-Moreno R, Cuesta-Geijo MA, Martinez-Romero C, Barrado-Gil L, Galindo I, Garcia-Sastre A, Alonso C. 2016. Antiviral role of IFITM proteins in African swine fever virus infection. PLoS One 11:e0154366. doi:10.1371/journal.pone.0154366. 27116236 PMC4846163

[14] Li C, Zheng HQ, Wang YF, Dong W, Liu YR, Zhang L, Zhang YM. 2019. Antiviral role of IFITM proteins in classical swine fever virus infection. Viruses 11:126. doi:10.3390/v11020126. PMC6409519 30704088

[15] Chen SL, Wang L, Chen JY, Zhang LL, Wang S, Goraya MU, Chi XJ, Na Y, Shao WH, Yang Z, Zeng XC, Chen SY, Chen JL. 2017. Avian interferon-inducible transmembrane protein family effectively restricts avian tembusu virus infection. Front Microbiol 8:672. doi:10.3389/fmicb.2017.00672. 28473814 PMC5397487

[16] Huang IC, Bailey CC, Weyer JL, Radoshitzky SR, Becker MM, Chiang JJ, Brass AL, Ahmed AA, Chi X, Dong L, Longobardi LE, Boltz D, Kuhn JH, Elledge SJ, Bavari S, Denison MR, Choe H, Farzan M. 2011. Distinct patterns of IFITM-mediated restriction of filoviruses, SARS coronavirus, and influenza A virus. PLoS Pathog 7:e1001258. doi:10.1371/journal.ppat.1001258. 21253575 PMC3017121

[17] Anafu AA, Bowen CH, Chin CR, Brass AL, Holm GH. 2013. Interferon-inducible transmembrane protein 3 (IFITM3) restricts reovirus cell entry. J Biol Chem 288:17261–17271. doi:10.1074/jbc.M112.438515. 23649619 PMC3682530

[18] Lin TY, Chin CR, Everitt AR, Clare S, Perreira JM, Savidis G, Aker AM, John SP, Sarlah D, Carreira EM, Elledge SJ, Kellam P, Brass AL. 2013. Amphotericin B increases influenza A virus infection by preventing IFITM3-mediated restriction. Cell Rep 5:895–908. doi:10.1016/j.celrep.2013.10.033. 24268777 PMC3898084

[19] Li K, Markosyan RM, Zheng YM, Golfetto O, Bungart B, Li MH, Ding SL, He YX, Liang C, Lee JC, Gratton E, Cohen FS, Liu SL. 2013. IFITM proteins restrict viral membrane hemifusion. PLoS Pathog 9:e1003124. doi:10.1371/journal.ppat.1003124. 23358889 PMC3554583

[20] Mudhasani R, Tran JP, Retterer C, Radoshitzky SR, Kota KP, Altamura LA, Smith JM, Packard BZ, Kuhn JH, Costantino J, Garrison AR, Schmaljohn CS, Huang IC, Farzan M, Bavari S. 2013. IFITM-2 and IFITM-3 but not IFITM-1 restrict Rift Valley fever virus. J Virol 87:8451–8464. doi:10.1128/JVI.03382-12. 23720721 PMC3719792

[21] Jia R, Pan QH, Ding SL, Rong LW, Liu SL, Geng YQ, Qiao WT, Liang C. 2012. The N-terminal region of IFITM3 modulates its antiviral activity by regulating IFITM3 cellular localization. J Virol 86:13697–13707. doi:10.1128/JVI.01828-12. 23055554 PMC3503121

[22] Jia R, Xu FW, Qian J, Yao YF, Miao CH, Zheng YM, Liu SL, Guo F, Geng YQ, Qiao WT, Liang C. 2014. Identification of an endocytic signal essential for the antiviral action of IFITM3. Cell Microbiol 16:1080–1093. doi:10.1111/cmi.12262. 24521078 PMC4065222

[23] Amini-Bavil-Olyaee S, Choi YJ, Lee JH, Shi MD, Huang IC, Farzan M, Jung JU. 2013. The antiviral effector IFITM3 disrupts intracellular cholesterol homeostasis to block viral entry. Cell Host Microbe 17:452–452. 10.1016/j.chom.2013.03.006 PMC3646482 23601107

[24] Yount JS, Karssemeijer RA, Hang HC. 2012. S-palmitoylation and ubiquitination differentially regulate interferon-induced transmembrane protein 3 (IFITM3)-mediated resistance to influenza virus. J Biol Chem 287:19631–19641. doi:10.1074/jbc.M112.362095. 22511783 PMC3365998

[25] Tartour K, Appourchaux R, Gaillard J, Nguyen XN, Durand S, Turpin J, Beaumont E, Roch E, Berger G, Mahieux R, Brand D, Roingeard P, Cimarelli A. 2014. IFITM proteins are incorporated onto HIV-1 virion particles and negatively imprint their infectivity. Retrovirology 11:103. doi:10.1186/s12977-014-0103-y. 25422070 PMC4251951

[26] Compton AA, Bruel T, Porrot F, Mallet A, Sachse M, Euvrard M, Liang C, Casartelli N, Schwartz O. 2014. IFITM proteins incorporated into HIV-1 virions impair viral fusion and spread. Cell Host Microbe 16:736–747. doi:10.1016/j.chom.2014.11.001. 25464829 PMC7104936

[27] Lunney JK, Fang Y, Ladinig A, Chen NH, Li YH, Rowland B, Renukaradhya GJ. 2016. Porcine reproductive and respiratory syndrome virus (PRRSV): pathogenesis and interaction with the immune system. Annu Rev Anim Biosci 4:129–154. doi:10.1146/annurev-animal-022114-111025. 26646630

[28] Bordet E, Blanc F, Tiret M, Crisci E, Bouguyon E, Renson P, Maisonnasse P, Bourge M, Leplat JJ, Giuffra E, Jouneau L, Schwartz-Cornil I, Bourry O, Bertho N. 2018. Porcine reproductive and respiratory syndrome virus type 1.3 Lena triggers conventional dendritic cells 1 activation and T helper 1 immune response without infecting dendritic cells. Front Immunol 9:2299. doi:10.3389/fimmu.2018.02299. 30333837 PMC6176214

[29] Adams MJ, Lefkowitz EJ, King AMQ, Harrach B, Harrison RL, Knowles NJ, Kropinski AM, Krupovic M, Kuhn JH, Mushegian AR, Nibert M, Sabanadzovic S, Sanfacon H, Siddell SG, Simmonds P, Varsani A, Zerbini FM, Gorbalenya AE, Davison AJ. 2017. Changes to taxonomy and the International Code of Virus Classification and Nomenclature ratified by the International Committee on Taxonomy of Viruses (2017). Arch Virol 162:2505–2538. doi:10.1007/s00705-017-3358-5. 28434098

[30] Rossow KD, Collins JE, Goyal SM, Nelson EA, Christopher-Hennings J, Benfield DA. 1995. Pathogenesis of porcine reproductive and respiratory syndrome virus infection in gnotobiotic pigs. Vet Pathol 32:361–373. doi:10.1177/030098589503200404. 7483210

[31] Van Breedam W, Delputte PL, Van Gorp H, Misinzo G, Vanderheijden N, Duan XB, Nauwynck HJ. 2010. Porcine reproductive and respiratory syndrome virus entry into the porcine macrophage. J Gen Virol 91:1659–1667. doi:10.1099/vir.0.020503-0. 20410315

[32] Kim HS, Kwang J, Yoon IJ, Joo HS, Frey ML. 1993. Enhanced replication of porcine reproductive and respiratory syndrome (PRRS) virus in a homogeneous subpopulation of MA-104 cell line. Arch Virol 133:477–483. doi:10.1007/BF01313785. 8257302

[33] Zhang A, Duan H, Li N, Zhao L, Pu F, Huang B, Wu C, Nan Y, Du T, Mu Y, Zhao Q, Sun Y, Zhang G, Hiscox JA, Zhou EM, Xiao S. 2017. Heme oxygenase-1 metabolite biliverdin, not iron, inhibits porcine reproductive and respiratory syndrome virus replication. Free Rad Biol Med 102:149–161. doi:10.1016/j.freeradbiomed.2016.11.044. 27908781

[34] Delputte PL, Vanderheijden N, Nauwynck HJ, Pensaert MB. 2002. Involvement of the matrix protein in attachment of porcine reproductive and respiratory syndrome virus to a heparinlike receptor on porcine alveolar macrophages. J Virol 76:4312–4320. doi:10.1128/jvi.76.9.4312-4320.2002. 11932397 PMC155060

[35] Guo LJ, Niu JW, Yu HD, Gu WH, Li R, Luo XL, Huang MM, Tian ZJ, Feng L, Wang Y. 2016. Correction for Guo et al., Modulation of CD163 expression by metalloprotease ADAM17 regulates porcine reproductive and respiratory syndrome virus entry. J Virol 90:5532–5532. doi:10.1128/JVI.00536-16. 27174637 PMC4934741

[36] Delputte PL, Van Breedam W, Delrue I, Oetke C, Crocker PR, Nauwynck HJ. 2007. Porcine arterivirus attachment to the macrophage-specific receptor sialoadhesin is dependent on the sialic acid-binding activity of the N-terminal immunoglobulin domain of sialoadhesin. J Virol 81:9546–9550. doi:10.1128/JVI.00569-07. 17567703 PMC1951444

[37] Van Gorp H, Van Breedam W, Delputte PL, Nauwynck HJ. 2009. The porcine reproductive and respiratory syndrome virus requires trafficking through CD163-positive early endosomes, but not late endosomes, for productive infection. Arch Virol 154:1939–1943. doi:10.1007/s00705-009-0527-1. 19885719

[38] Desai TM, Marin M, Chin CR, Savidis G, Brass AL, Melikyan GB. 2014. IFITM3 restricts influenza A virus entry by blocking the formation of fusion pores following virus-endosome hemifusion. PLoS Pathog 10:e1004048. doi:10.1371/journal.ppat.1004048. 24699674 PMC3974867

[39] Liscum L, Sturley SL. 2004. Intracellular trafficking of Niemann-Pick C proteins 1 and 2: obligate components of subcellular lipid transport. Biochim Biophys Acta 1685:22–27. doi:10.1016/j.bbalip.2004.08.008. 15465423

[40] Yu J, Li M, Wilkins J, Ding S, Swartz TH, Esposito AM, Zheng YM, Freed EO, Liang C, Chen BK, Liu SL. 2015. IFITM proteins restrict HIV-1 infection by antagonizing the envelope glycoprotein. Cell Rep 13:145–156. doi:10.1016/j.celrep.2015.08.055. 26387945 PMC4602366

[41] Guo R, Katz BB, Tomich JM, Gallagher T, Fang Y. 2016. Porcine reproductive and respiratory syndrome virus utilizes nanotubes for intercellular spread. J Virol 90:5163–5175. doi:10.1128/JVI.00036-16. 26984724 PMC4859731

[42] Suddala KC, Lee CC, Meraner P, Marin M, Markosyan RM, Desai TM, Cohen FS, Brass AL, Melikyan GB. 2019. Interferon-induced transmembrane protein 3 blocks fusion of sensitive but not resistant viruses by partitioning into virus-carrying endosomes. PLoS Pathog 15:e1007532. doi:10.1371/journal.ppat.1007532. 30640957 PMC6347298

[43] Kuhnl A, Musiol A, Heitzig N, Johnson DE, Ehrhardt C, Grewal T, Gerke V, Ludwig S, Rescher U. 2018. Late endosomal/lysosomal cholesterol accumulation is a host cell-protective mechanism inhibiting endosomal escape of influenza A virus. mBio 9:e01345-18. doi:10.1128/mBio.01345-18. 30042202 PMC6058292

[44] John SP, Chin CR, Perreira JM, Feeley EM, Aker AM, Savidis G, Smith SE, Elia AEH, Everitt AR, Vora M, Pertel T, Elledge SJ, Kellam P, Brass AL. 2013. The CD225 domain of IFITM3 is required for both IFITM protein association and inhibition of influenza A virus and dengue virus replication. J Virol 87:7837–7852. doi:10.1128/JVI.00481-13. 23658454 PMC3700195

[45] Yount JS, Moltedo B, Yang YY, Charron G, Moran TM, Lopez CB, Hang HC. 2010. Palmitoylome profiling reveals S-palmitoylation-dependent antiviral activity of IFITM3. Nat Chem Biol 6:610–614. doi:10.1038/nchembio.405. 20601941 PMC2928251

[46] Tartour K, Nguyen XN, Appourchaux R, Assil S, Barateau V, Bloyet LM, Burlaud Gaillard J, Confort MP, Escudero-Perez B, Gruffat H, Hong SS, Moroso M, Reynard O, Reynard S, Decembre E, Ftaich N, Rossi A, Wu N, Arnaud F, Baize S, Dreux M, Gerlier D, Paranhos-Baccala G, Volchkov V, Roingeard P, Cimarelli A. 2017. Interference with the production of infectious viral particles and bimodal inhibition of replication are broadly conserved antiviral properties of IFITMs. PLoS Pathog 13:e1006610. doi:10.1371/journal.ppat.1006610. 28957419 PMC5619827

[47] Sharma A, McLaughlin RN, Jr, Basom RS, Kikawa C, OhAinle M, Yount JS, Emerman M, Overbaugh J. 2019. Macaque interferon-induced transmembrane proteins limit replication of SHIV strains in an Envelope-dependent manner. PLoS Pathog 15:e1007925. doi:10.1371/journal.ppat.1007925. 31260493 PMC6625738

[48] Zhao XS, Guo F, Liu F, Cuconati A, Chang JH, Block TM, Guo JT. 2014. Interferon induction of IFITM proteins promotes infection by human coronavirus OC43. Proc Natl Acad Sci U S A 111:6756–6761. doi:10.1073/pnas.1320856111. 24753610 PMC4020042

[49] Wensvoort G, Terpstra C, Pol JM, ter Laak EA, Bloemraad M, de Kluyver EP, Kragten C, van Buiten L, den Besten A, Wagenaar F. 1991. Mystery swine disease in The Netherlands: the isolation of Lelystad virus. Vet Q 13:121–130. doi:10.1080/01652176.1991.9694296. 1835211

[50] Wang CB, Huang BC, Kong N, Li QY, Ma YP, Li ZJ, Gao JM, Zhang C, Wang XP, Liang C, Dang L, Xiao SQ, Mu Y, Zhao Q, Sun Y, Almazan F, Enjuanes L, Zhou EM. 2013. A novel porcine reproductive and respiratory syndrome virus vector system that stably expresses enhanced green fluorescent protein as a separate transcription unit. Vet Res 44:104. doi:10.1186/1297-9716-44-104. 24176053 PMC4176086

[51] Zhu X, He ZJ, Yuan J, Wen WT, Huang X, Hu YW, Lin CJ, Pan J, Li R, Deng HJ, Liao SW, Zhou R, Wu JH, Li J, Li MF. 2015. IFITM3-containing exosome as a novel mediator for anti-viral response in dengue virus infection. Cell Microbiol 17:105–118. doi:10.1111/cmi.12339. 25131332 PMC7162390

[52] Shaw ML, Stone KL, Colangelo CM, Gulcicek EE, Palese P. 2008. Cellular proteins in influenza virus particles. PLoS Pathog 4:e1000085. doi:10.1371/journal.ppat.1000085. 18535660 PMC2390764

[53] Sakai T, Ohuchi M, Imai M, Mizuno T, Kawasaki K, Kuroda K, Yamashina S. 2006. Dual wavelength imaging allows analysis of membrane fusion of influenza virus inside cells. J Virol 80:2013–2018. doi:10.1128/JVI.80.4.2013-2018.2006. 16439557 PMC1367152

[54] Lakadamyali M, Rust MJ, Babcock HP, Zhuang X. 2003. Visualizing infection of individual influenza viruses. Proc Natl Acad Sci U S A 100:9280–9285. doi:10.1073/pnas.0832269100. 12883000 PMC170909

