# Supplementary material for: Interferon-Induced Transmembrane Protein 3 Is a Virus-Associated Protein Which Suppresses Porcine Reproductive and Respiratory Syndrome Virus Replication by Blocking Viral Membrane Fusion
Source: J Virol. 2020 Nov 23;94(24):e01350-20. doi: 10.1128/JVI.01350-20 (PMC7925183; doi:10.1128/JVI.01350-20)
Supplement: Supplemental file 1 [file JVI.01350-20-s0003.pdf]

Movie S1 Example of PRRSV intercellular transmission when Marc-145-Vector cells are used as both donor cells and target cells. Marc-145-Vector cells were infected with 0.1 MOI of GFP-PRRSV for 48 h. Cells were digested and counted as donor cells. $5x10^5$ donor cells (green) were co-cultured with $5x10^5$ Marc-145-Vector-RFP cells (red, target cells) for 2 h at 37˚C, followed by sorting using flow cytometry. About $4x10^5$ target cells were plated in 35 mm culture dishes and continued to incubate for 12 h at 37˚C, then time-lapse video imaging was conducted. Transmission and fluorescence images were taken every 5 min up to 60 hpi at 37˚C, 5% CO_2 using a Nikon Biostation IMQ.

Movie S2 Example of PRRSV intercellular transmission when Marc-145-IFITM3-flag cells are used as both donor cells and target cells. Marc-145-IFITM3-flag-RFP cells were infected with 0.1 MOI of GFP-PRRSV for 48 h. Cells were digested and counted as donor cells. $5x10^5$ donor cells (green) were cocultured with $5x10^5$ Marc-145-IFITM3-flag-RFP cells (red, target cells) for 2 h at 37˚C, followed by sorting using flow cytometry. About $4x10^5$ target cells were plated in 35 mm culture dishes and continued to incubate for 12 h at 37˚C, then time-lapse video imaging was conducted. Transmission and fluorescence images were taken every 5 min up to 60 hpi at 37˚C, 5% CO_2 using a Nikon Biostation IMQ.
